# ﻿Discovering diversity of Central Asian and Himalayan *Epeorus* (*Caucasiron*) mayflies (Ephemeroptera, Heptageniidae) using DNA barcoding and morphology

**DOI:** 10.3897/zookeys.1234.141196

**Published:** 2025-04-09

**Authors:** Ľuboš Hrivniak, Pavel Sroka, Roman J. Godunko, Alexander V. Martynov, Dmitry M. Palatov, Jindřiška Bojková

**Affiliations:** 1 Department of Botany and Zoology, Faculty of Science, Masaryk University, Kotlářská 2, 61137 Brno, Czech Republic; 2 Biology Centre of the Czech Academy of Sciences, Institute of Entomology, Branišovská 31, 370 05 České Budějovice, Czech Republic; 3 Department of Invertebrate Zoology and Hydrobiology, Faculty of Biology and Environmental Protection, University of Lodz, Banacha 12/16, 90237 Łódź, Poland; 4 National Museum of Natural History, National Academy of Sciences of Ukraine, Bohdan Khmelnytsky str., 15, 01030, Kyiv, Ukraine; 5 Independent researcher

**Keywords:** Aquatic insects, integrative taxonomy, mountains, species delimitation

## Abstract

The mayflies of the genus Epeorus Eaton, 1881 subgenus Caucasiron Kluge, 1997 are distributed from the eastern Mediterranean to the mountains of south-west China. In contrast to the Caucasus, the Mediterranean and Irano-Anatolian regions, where E. (Caucasiron) represents one of the most extensively studied mayfly taxa, the species diversity in the more eastern mountains of Asia has been studied only sporadically. In this study, the species diversity of E. (Caucasiron) from the mountains of Central Asia (Pamir, Tian Shan) and the western part of the Himalayas was analysed using DNA barcoding and the morphology of larvae and adults. The distance- and phylogenetic tree-based molecular species delimitation analyses revealed five E. (Caucasiron) species occurring in the study area. Three of them did not correspond morphologically to any known species of the genus *Epeorus*. These species were described herein as E. (C.) himalayensis Hrivniak & Sroka, **sp. nov.**, E. (C.) lanceolatus Hrivniak & Sroka, **sp. nov.** and E. (C.) lineatus Hrivniak & Sroka, **sp. nov.** All new species were compared with other representatives of the subgenus and other related species of the genus *Epeorus*, and appropriate morphological diagnostic characters were provided. Morphological revision, main diagnostic characters, and information on the distribution of E. (C.) guttatus Braasch & Soldán, 1979 and two other potentially related *Epeorus* species from the area, *E.psi* Eaton, 1885 and *E.suspicatus* (Braasch, 2006), are also given.

## ﻿Introduction

**Figure 1.**
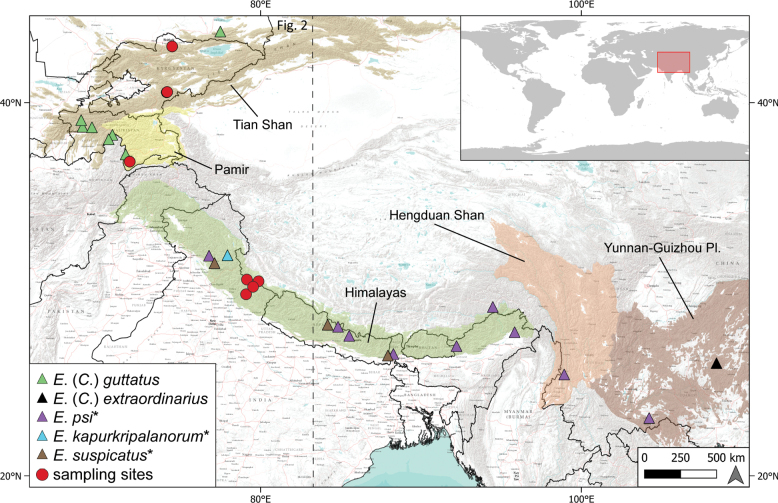
Distribution of studied *Epeorus* spp. across Asian mountains. Triangles refer to localities in the literature; our sampling sites are marked with red dots. A species name marked with an asterisk indicates the unclear systematic position of the species within the genus *Epeorus*. Dashed line represents the frame of the detailed map in Fig. 2. Boundaries of mountain ranges were adopted from Snethlage et al. (2022).

The genus Epeorus Eaton, 1881, subgenus Caucasiron Kluge, 1997, represents a charismatic group of mountain mayflies distributed in the Palaearctic (Hrivniak et al. 2020a). Its highest diversity is to be found in the Caucasus region, where it represents one of the most species-rich groups of mayflies (Hrivniak et al. 2024). Its larvae inhabit rapids of montane and submontane streams, as well as rivers with stony substrates, and are among the most important indicators of high water quality (Türkmen 2023). The distribution range of E. (Caucasiron) can be divided into two separate areas. The western part includes the Eastern Mediterranean (Samos and Cyprus Islands), the Caucasus and the Irano-Anatolian mountain ranges (Hrivniak et al. 2020b), while the eastern part includes the mountains of Central Asia, the Himalayas, and the mountains of south-west China (Fig. 1). The species diversity of the western part of the area was already intensively studied from 1938 to 1981 (Tshernova 1938; Sinitshenkova 1976; Braasch 1978, 1979, 1980a, 1983a; Braasch and Soldán 1979; Braasch and Zimmerman 1979) and altogether ten species were described based on morphology. Since 2017, a further eight species have been described using an integrative taxonomic approach (Hrivniak et al. 2017, 2019, 2020a, 2021, 2022, 2024), when the analysis of morphological traits proposed by Braasch and Soldán (1979) and Hrivniak et al. (2020b) were combined with the DNA barcoding (sequences of cytochrome c oxidase subunit I; hereafter COI) and molecular species delimitation (e.g. Fujisawa and Barraclough 2013; Puillandre et al. 2021). To date, the research has resulted in 18 described species (listed in Hrivniak et al. 2020b, 2024), a comprehensive DNA barcoding dataset of all species (deposited in NCBI GenBank, https://www.ncbi.nlm.nih.gov/genbank) and a guide for the identification of larvae to the species level (Hrivniak et al. 2020b, 2024).

In contrast to the western part of the area, only two species reliably attributed to E. (Caucasiron) are known from the eastern part of the area, namely E. (C.) guttatus (Braasch & Soldán, 1979) distributed in the mountains of Central Asia (Tian Shan and Pamir) (Braasch and Soldán 1979; Kluge 2015) and E. (C.) extraordinarius Chen, Wang & Zhou, 2010 known from south-west China (Yunnan-Guizhou Plateau) (Chen et al. 2010; Ma and Zhou 2022) (Fig. 1). The recent study by Vasanth et al. (2021) summarised the diversity and distribution of the genus *Epeorus* in India and placed three additional species in the subgenus E. (Caucasiron). These included *E.psi* Eaton, 1885 distributed in the Himalayas and Mountains of south-west China (Braasch 1980b, 1981; Vasanth et al. 2021; Ma and Zhou 2022), and *E.kapurkripalanorum* (Braasch, 1983) and *E.suspicatus* (Braasch, 2006), both of which are known from the Himalayas (Kapur and Kripalani 1963; Braasch 2006a) (Fig. 1). However, this was based solely on larval morphology or on original descriptions of larvae, but not on the analysis of molecular data or the examination of adults, which are essential for the assignment of species to species groups/subgenera (Braasch 2006a). Therefore, the subgeneric classification of these species within the genus *Epeorus* remains uncertain (Kluge 2004; Braasch 2006a) and a reliable taxonomic revision is required to clarify their systematic position. In this study, we retain the assignment of *E.psi*, *E.kapurkripalanorum* and *E.suspicatus* to the genus level, while we consider the assignment to the subgenus E. (Caucasiron) to be uncertain.

Considering the geographical extent and topographical complexity of the eastern part of the area, more species of E. (Caucasiron) can be expected there. Therefore, we aim to investigate its species diversity in Central Asia and the Himalayas using an integrative taxonomic approach that has proven successful in the species delimitation of E. (Caucasiron) mayflies in the western part of the area. Since species identification based on the original descriptions is often complicated and inaccurate, we also aim to revise the morphology of insufficiently described species.

This study summarises the recent knowledge on E. (Caucasiron) from the eastern part of its range, focusing on the mountains of Central Asia (Tian Shan and Pamir) and the western part of the Himalayas (Fig. 1). The main objectives are (i) to study the species diversity of the subgenus in Central Asia and the Himalayas using molecular species delimitation tools and morphology, (ii) to describe the morphology of new species and provide their key diagnostic characters, and (iii) to provide basic information on their distribution and habitat requirements. In addition, we provide information on larval morphology, diagnostic characters, and distribution of three species of the genus *Epeorus* known from the area, E. (C.) guttatus, *E.suspicatus*, and *E.psi*.

## ﻿Materials and methods

The specimens of E. (Caucasiron) examined in this study were collected in Tajikistan, Kyrgyzstan, and India in 2016–2018. The larvae were collected by hand net and the winged stages were reared from larvae in the field. The material was preserved in 96% EtOH and stored in the laboratory at -20 °C. The col­lection is deposited at the Zoological Survey of India (**ZSI**), Kolkata (The Ministry of Environment, Forest and Climate Change, India), Biology Centre of the Czech Academy of Sciences, Institute of Entomology, České Budějovice, Czech Republic (**IECA**) and National Museum of Natural History, National Academy of Sciences of Ukraine (**NMNH NASU**). Other species used for morphological comparison were obtained from the IECA. These included all species from the Caucasus, the Mediterranean region and Irano-Anatolian region, *E.psi* and the type material (paratype) of E. (C.) guttatus (Braasch & Soldán, 1979). The type material (holotype and paratypes) of *E.suspicatus* (Braasch, 2006) was obtained from the State Museum of Natural History, Stuttgart, Germany (**SMNS**).

### ﻿Morphological examination and terminology

Parts of specimens were mounted on microscopic slides using HydroMatrix (MicroTech Lab, Graz, Austria) mounting medium. To remove the muscle tissue for an investigation of the cuticular structures, specimens were left overnight in a 10% solution of NaOH prior to slide mounting. Drawings were made using a stereomicroscope Olympus SZX7 and a microscope Olympus BX41, both equipped with a drawing tube. Photographs were obtained using Leica DFC450 camera fitted with macroscope Leica Z16 APO. Photographs were stacked in Helicon Focus v. 5.3. All photographs were subsequently enhanced with Adobe Photoshop v. CS5. Specimens assignable to already described species were identified using original descriptions/redescriptions of individual species (Kapur and Kripalani 1963; Braasch 1980b, 2006a; Chen et al. 2010; Kluge 2015; Vasanth et al. 2021) and comparative material. Adults were associated with larvae by rearing in the field and analyses of COI. Morphological traits for the description of larvae and adults were adopted from Braasch (1980a) and Hrivniak et al. (2020).

A term “late instar larva“ refers to the larva with well-developed wing pads (as on Fig. 3A) or the larva with body length exceeding 10 mm without cerci. A term “extralimital species” used in sections “Affinities” includes species from the western part of E. (Caucasiron) range (i.e., the Caucasus, Mediterranean and Irano-Anatolian regions). The systematic classification follows the concept of Kluge (1997, 2015), where several subgenera within the genus *Epeorus* are recognised. Species with unclear subgeneric position are classified to the genus level only.

### ﻿DNA extraction, PCR, sequencing, and alignment

Total genomic DNA of 25 specimens was extracted from legs using the DEP-25 DNA Extraction Kit (Top-Bio) or DNeasy Blood & Tissue Kit (QIAGEN). COI was sequenced according to Hrivniak et al. (2017). COI sequences of other E. (Caucasiron) species used in the species delimitation analyses were obtained from Hrivniak et al. (2020a, 2021, 2022, 2024) and Ma and Zhou (2022). The final dataset contained 123 specimens, all species from the Caucasus, Mediterranean, and Irano-Anatolian regions, and a single species from south-west China (E. (C.) extraordinarius). Sequences were assembled in Jalview v. 2 (Waterhouse et al. 2009) and aligned in the same software using the Mafft v. 7 plugin (Katoh and Standley 2013). Newly obtained sequences are deposited in GenBank with accession numbers PV330270–PV330294.

### ﻿Molecular species delimitation

Molecular delimitation of individual species was performed using the single threshold General Mixed Yule Coalescent model (GMYC, Pons et al. 2006; Fujisawa and Barraclough 2013) and the Assemble Species by Automatic Partitioning (ASAP; Puillandre et al. 2021). The COI gene tree for GMYC model was reconstructed using BEAST v. 2.6.7. (Bouckaert et al. 2019) with settings described in Hrivniak et al. (2020a). Two analyses were run on CIPRES Science Gateway (Miller et al. 2010) for 100 million generations sampled every 10,000 generations. Convergence and effective sample size (ESS > 200) were verified using Tracer v. 1.7. (Rambaut et al. 2018). The first 10% of trees from each run were discarded as burn-in. Files from both independent runs were combined using LogCombiner v. 2.6.7. The maximum clade credibility tree was constructed from 18,000 trees using TreeAnnotator v. 1.8.4. with default settings. GMYC model was performed at https://species.h-its.org/gmyc/. ASAP was performed at https://bioinfo.mnhn.fr/abi/public/asap/. The input dataset was the aligned fasta file and a simple pairwise genetic distances were selected. Inter- and intraspecific pairwise genetic distances were calculated in MEGA X (Kumar et al. 2018).

## ﻿Results and discussion

### ﻿Molecular and morphological species delimitation

**Figure 2.**
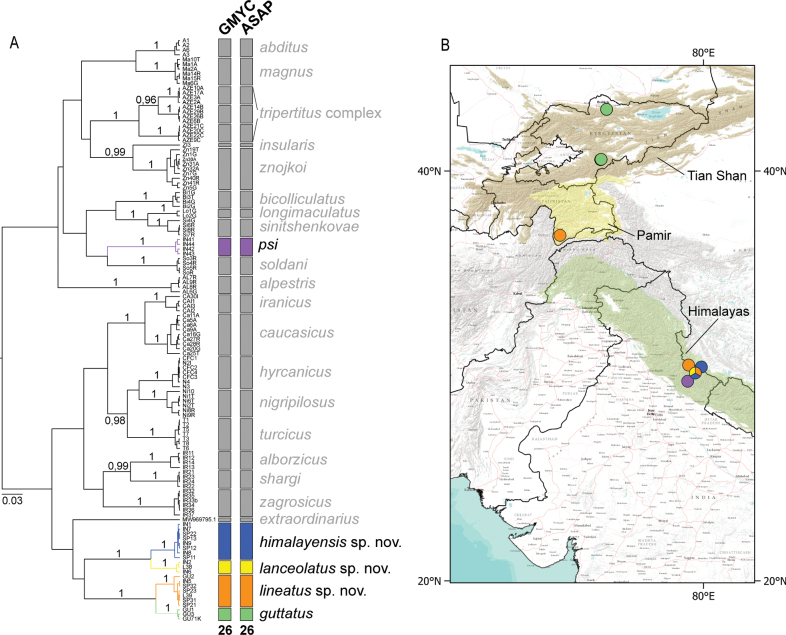
**A**COI gene tree generated in BEAST 2 and results of GMYC and ASAP species delimitation analyses. Numbers on the tree branches refer to the posterior probability. Species delimited from our sampling sites in Central Asia and the Himalayas are highlighted and colored **B** occurrence of delimited species in the study area (colors correspond to **A**).

The final alignment contained 631 base pairs and 226 variable positions, from which 211 were parsimony informative. The GMYC model revealed 26 species units (confidence interval: 23–31). The most probable scenario of ASAP species delimitation (ASAP score of partition 1: 1.50; threshold distance: 0.043582) was congruent with GMYC model (Fig. 2). All species clusters were supported by posterior probability (> 0.95) from BEAST 2. Specimens from Central Asia and the Himalayas were delimited into five species units. Two of them morphologically corresponded to E. (C.) guttatus and *E.psi.* The remaining three species units did not correspond to any previously known E. (Caucasiron) species. They also differed from similar species with unclear subgeneric attribution occurring in the same area, *E.kapurkripalanorum* and *E.suspicatus*. Therefore, we have named the three new species E. (C.) himalayensis sp. nov., E. (C.) lanceolatus sp. nov., and E. (C.) lineatus sp. nov. and their morphology is described below.

**Figure 3.**
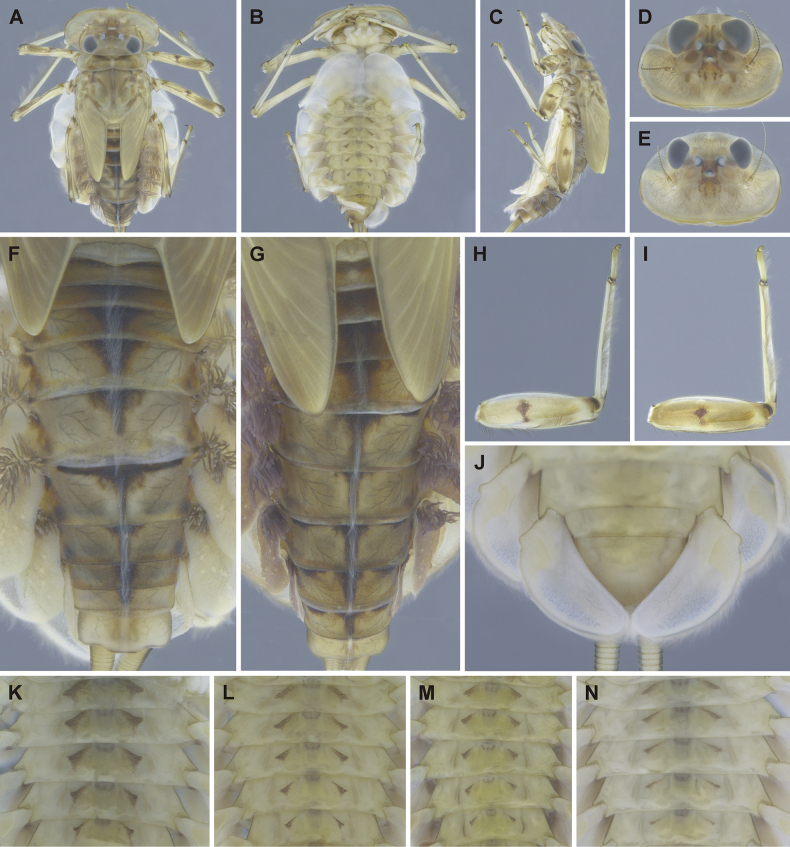
Epeorus (Caucasiron) himalayensis sp. nov., larva **A** habitus in dorsal view **B** habitus in ventral view **C** habitus in lateral view **D** head of male in dorsal view **E** head of female in dorsal view **F, G** coloration of abdominal terga **H, I** middle leg in dorsal view **J** distal part of abdomen in ventral view **K–M** coloration of abdominal sterna.

Interspecific pairwise genetic distances of the dataset restricted to species from Central Asia, the Himalayas and south-west China ranged from 5.07 to 18.07% and maximum intraspecific distances reached 1.59%. Interspecific pairwise genetic distances of morphologically well-defined species distributed in the Caucasus, Mediterranean and Irano-Anatolian region ranged from 5.24 to 16.03% and maximum intraspecific distances reached 3.53%.

The newly proposed species E. (C.) himalayensis sp. nov. differed from all other species included in the analysis by 6.25–17.49% (E. (C.) lanceolatus sp. nov.: 6.25–6.83% and *E.psi*: 16.51–17.49%), E. (C.) lineatus sp. nov. differed by 5.07–18.07% (E. (C.) guttatus: 5.07–5.71% and *E.psi*: 16.83–18.07%), and E. (C.) lanceolatus sp. nov. differed by 6.25–17.27% (E. (C.) himalayensis sp. nov.: 6.25–6.83% and *E.psi*: 16.32–17.27%).

### ﻿Descriptions of the new species

All species described below were assigned to the subgenus Caucasiron within the genus *Epeorus* based on the following morphological characters: larva: i) presence of a projection on the costal margin of gill plates II–VII, and ii) mediodorsal directed hair-like setae along the anterior margin of the head; male imago: iii) penis lobes cylindrical, without latero-apical spines, and iv) median titillators well developed. The holotypes are deposited in ZSI, paratypes in IECA and NMNH NASU.

#### ﻿ Epeorus (Caucasiron) himalayensis

Taxon classificationAnimaliaEphemeropteraHeptageniidae

Hrivniak & Sroka sp. nov.

691B9F9C-6D9B-5720-A574-0E797BF2EC19

https://zoobank.org/8A4B0D7A-B2FD-42B8-85DB-87FCFC1D2140

Figs 3 , 4 , 5 , 6

##### Type material.

***Holotype***: • male larva: India: Uttarakhand Pradesh, vicinity of Pandukeshwar village, left tributary of Alakananda River, 2099 m a.s.l., 30°38.901'N, 79°32.108'E (codes: IND2018/7; 39Gang); 9–11.05.2018, Martynov A.V., Palatov D.M. leg. ***Paratypes***: • 12 larvae (barcoded specimens: IN1, SP11 - labrum and mandibular incisors mounted on slide, SP12), 4 male imagoes (reared from larvae; barcoded specimens: IN7 - genitalia and larval exuvia mounted on slide, IN8, IN9), 3 female imagoes (reared from larva; two larval exuviae mounted on slide), 1 male subimago (reared from larva): same data as holotype. • 2 larvae (barcoded specimens: SP13, SP22): India: Uttarakhand Pradesh, vicinity of Lambagad village, Alaknanda River, 1998 m a.s.l.; 30°38.64198'N, 79°32.02500'E (codes: IND2018/8; 40Gang); 9–11.05.2018, Martynov A.V., Palatov D.M. leg.

##### Etymology.

The species name *himalayensis* (Latin) refers to the distribution in the Himalayas.

##### Description of larva.

General coloration yellowish brown with dark brown maculation (Fig. 3). Body length (BL) of late-instar larvae: 15.0 mm (female; *n* = 1), 11.8–13.1 mm (male; *n* = 3). Length of cerci approximately 1.2 × body length.

**Figure 4.**
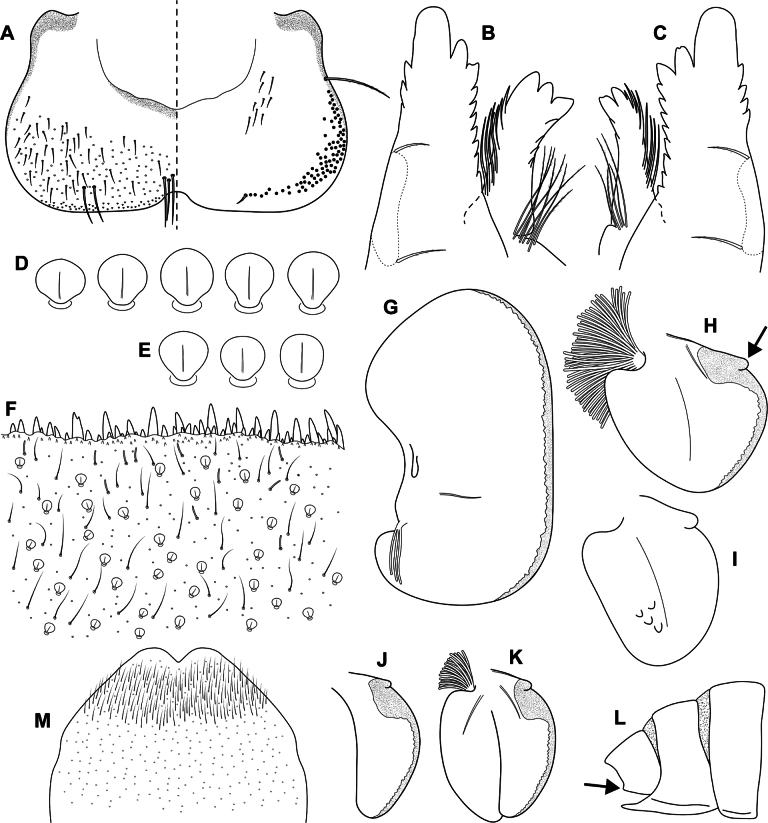
Epeorus (Caucasiron) himalayensis sp. nov., larva **A** labrum, left half in dorsal view, right half in ventral view (black dots correspond to setae along antero-lateral margin) **B** incisors of left mandible **C** incisors of right mandible (both flattened on slide; dashed polygons correspond to area covered by setae) **D** setae on dorsal surface of femora **E** setae on surface of tergum VII **F** surface and posterior margin of abdominal tergum VII **G** gill I **H** gill III (arrow shows projection on costal margin) **I** gill plate VI in dorsal view **J** gill plate VII in ventral view **K** gill VII (flattened on slide) **L** abdominal segments VIII–X in lateral view (arrow shows posterolateral projection of tergum X) **M** sternum IX of female. Drawn from late instar larvae and last instar larval exuvium.

***Head*.** Shape trapezoidal, slightly rounded (Fig. 3D, E). Head dimensions of late-instar larvae: length 3.3 mm, width 5.0 mm in female; length 2.5–2.6 mm, width 4.0–4.1 mm in male. Width/length ratio: 1.48–1.57 (female; *n* = 8), 1.48–1.64 (male; *n* = 8). Coloration pattern of dorsal surface consists of: i) paired stripe-like and rounded maculae along epicranial suture, ii) pair of triangular (or blurred) macula near inner edges of eyes, iii) pair of rounded maculae ventrally to lateral ocelli, iv) pale stripes extending from lateral ocelli to lateral edges of head, v) rectangular macula between ocelli, vi) stripe-like and rounded maculae ventrally to median ocellus. Antennae yellowish brown, scapus and pedicellus darkened (Fig. 3D, E). Dorsal surface covered with short rounded spatulate setae (as on abdominal terga; Fig. 4E), fine hair-like setae and stick-like setae. Sparse longer and fine hair-like setae located posteriorly to eyes.

***Mouthparts*.** Labrum (Fig. 4A) widened anteriorly; anterior margin slightly rounded or nearly straight. Lateral angles rounded. Dorsal surface covered with setae of different size, 4–6 longer bristle-like setae located antero-medially and two bristles antero-laterally (Fig. 4A, left half). Epipharynx with longer, slightly plumose bristles situated along lateral to anterior margin, cluster of fine, hair-like setae medially (not figured), and group of 6–12 setae of various size (Fig. 4A, right half). Outer incisors of both mandibles with three apical teeth; outer tooth blunt in both mandibles. Inner incisor of left mandible with three apical teeth (Fig. 4B), right inner incisor bifurcated (Fig. 4C).

***Thorax*.** Prothorax anteriorly narrowed, lateral edges slightly rounded. Metanotum with small blunt posterior-median projection. Dorsal surface covered with hair-like setae, stick-like setae and short rounded spatulate setae (as on abdominal terga and head). Sparse longer, hair-like setae along pro-, meso- and metanotal suture.

***Legs*.** Coloration on Fig. 3H, I. Femora with median hypodermal spot, often transversely extended. Base and apex of femora darkened; patella-tibial suture darkened; tarsi proximally and distally darkened. Dorsal surface of femora covered by short rounded spatulate setae (Fig. 4D), hair-like setae and sparsely distributed stick-like setae. Dorsal edge of femora with blade-like setae. Dorsal margin of tibiae and tarsi with row of dense hair-like setae; ventral margin with irregular row of distally accumulated spines. Tarsal claws with two or three denticles.

***Abdominal terga*.** Color pattern of abdominal terga consists of transversal stripe along anterior margin of terga I–IX (X), medially extending to: i) triangular or blurred macula on terga II–IV and ii) triangular or T-shaped macula on terga V–IX (median macula on terga VIII and IX widened) (Fig. 3F, G). Pair of short stripes or spots present antero-laterally to median maculae. Lateral margins with oblique stripe-like maculae on terga I–IX (often dorso-posteriorly extended). Denticles along posterior margin of terga dense, irregular, and pointed (Fig. 4F). Surface of terga covered with hair-like setae, stick-like setae and rounded spatulate setae (Fig. 4E, F). Tergum X with short posterolateral projections (Fig. 4L, arrow). Terga with longitudinal median row of hair-like setae.

More or less developed posteromedian spine (best expressed on terga VII–IX as on Fig. 11M, N) were observed in larvae of BL 6.0–8.2 mm (barcoded specimens SP12, SP13, SP22). Tergal spines were not observed in late instar larvae and last instar larval exuvia from reared adults (barcoded specimens: IN1, IN7, IN8, IN9).

***Abdominal sterna*.** Yellowish, with a pattern consisting of more or less defined triangular maculae (Fig. 3K–N). Nerve ganglia darkened. Sternum IX of female with V-shaped median emargination and numerous hair-like setae (Fig. 4M).

***Gills*.** Dorsal surface of gill plate I yellowish and of gill plates II–VII greyish on anterior half, brownish on posterior half. Ventral margin of all gill plates yellowish brown, sometimes pinkish. Projection on gill plate III well developed (Fig. 4H, arrow). Gill plate VII narrow (in natural position of ventral view, Figs 3J, 4J). Dorsal margin of gill plates IV–VII with more or less developed papillae; best expressed on gill plates VI–VII (Fig. 4I).

***Cerci*.** Yellowish brown, basally darkened.

##### Description of male imago.

**Figure 5.**
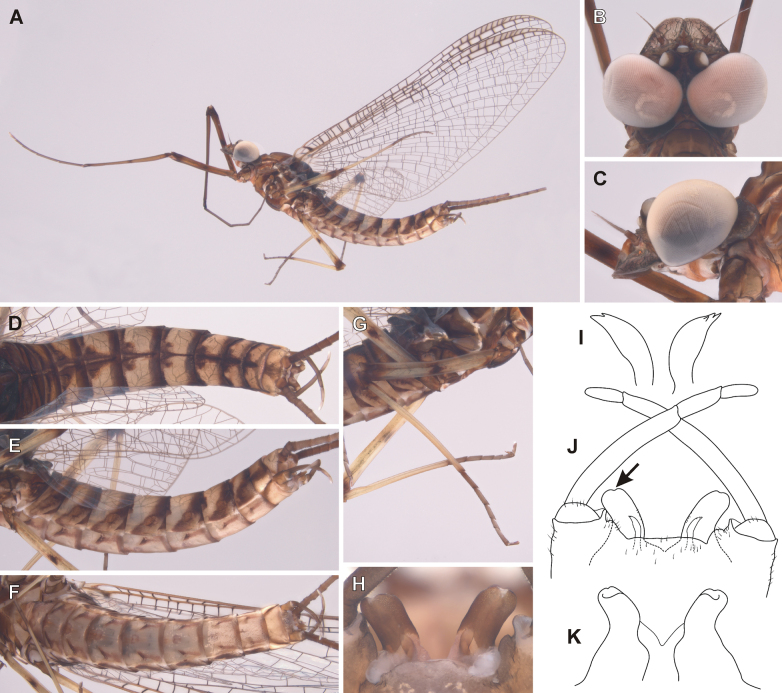
Epeorus (Caucasiron) himalayensis sp. nov., male imago **A** habitus in lateral view **B** head in dorsal view **C** head in lateral view **D** abdomen in dorsal view **E** abdomen in lateral view **F** abdomen in ventral view **G** middle leg in dorsal view **H** penis in ventral view **I** titillators **J** male genitalia in ventral view (arrow points on shallow medio-apical emargination) **K** penis in dorsal view.

General coloration yellowish brown with dark brown maculation (Fig. 5A). Body length 11.5–13.0 mm (*n* = 2); length of cerci approximately 2 × body length. Length of fore wings 14.0–15.3 mm, hind wings 4.5–5.1 mm.

***Head*.** Frons yellowish brown; frontal fold dark brown. Antennae yellowish; scapus and pedicellus darkened. Ocelli basally blackish, apically whitish. Compound eyes greyish brown, basally darkened (Fig. 5B, C). Compound eyes not touching each other (distance between eyes 0.10–0.66 of median ocellus; *n* = 3) or touching each other (*n* = 1).

***Thorax*.** Pronotum dark brown; meso- and metathorax yellowish brown with dark brown maculation. Dorsal surface of mesothorax yellowish brown, median longitudinal suture darkened. Mesothoratic fucasternum yellowish brown to brown. Metathorax with blunt posteromedian projection.

Wing membrane colorless. Veins dark brown, basally paler. Pterostigmatic area cloudy, with simple cross veins. Costal brace dark brown (Fig. 5A). Hind wings with short triangular costal projection.

Femora basally and apically darkened, median spot present. Tibiae basally darkened; claws dark brown (Fig. 5A, G). Fore legs darker than middle and hind legs. One claw blunt, one claw pointed.

***Abdomen*.** Color pattern of abdominal terga as described in larva. Tergum X with median macula (Fig. 5D). Lateral margins with oblique stripe-like maculae on terga I–IX extending dorso-posteriorly, forming transversal stripe-like macula along posterior margin of terga (Fig. 5D, E). Abdominal sterna with narrow triangular maculae (Fig. 5F). Styliger yellowish brown; medially slightly convex and sparsely covered by hair-like setae (Fig. 5H, J). Forceps brown or yellowish, apically paler. Penis lobes brown and basally paler or yellowish, with shallow medio-apical notch (Fig. 5H, J, arrow), and short spine-like setae on interior edges. Titillators well developed and apically serrated (Fig. 5H, I). Titillators reach 0.30–0.44 of respective penis lobe length.

***Cerci*.** Yellowish, basally darkened.

##### Description of female imago.

**Figure 6.**
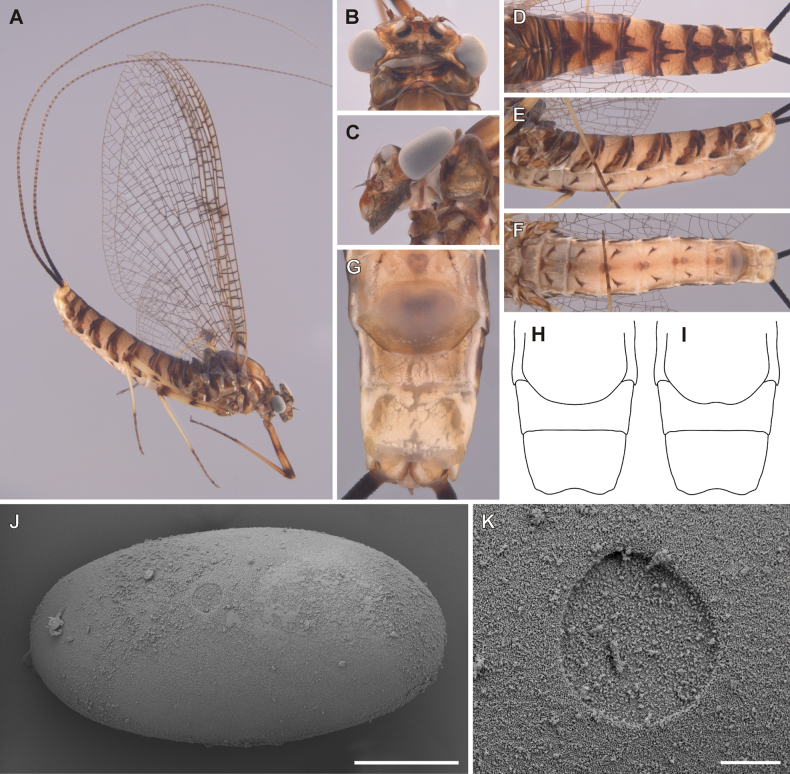
Epeorus (Caucasiron) himalayensis sp. nov., female imago and egg **A** habitus in lateral view **B** head in dorsal view **C** head in lateral view **D** abdomen in dorsal view **E** abdomen in lateral view **F** abdomen in ventral view **G–I** subgenital and subanal plates **J** egg **K** detail of micropyle. Scale bars: 5 μm (**K**); 50 μm (**J**).

General coloration yellowish brown with dark brown maculation (Fig. 6A). Body length 13.0–15.5 mm (*n* = 2); length of cerci 2.3 × body length. Length of fore wings 18.2–19.5 mm, hind wings 5.6–6.3 mm.

***Head*.** Frons yellowish brown; frontal fold brownish. Antennae yellowish; scapus and pedicellus darkened. Ocelli basally blackish, apically whitish. Eyes greyish (Fig. 6B, C).

***Thorax*.** Coloration as described in male imago. Wing membrane colorless (area around bullae sometimes darkened; Fig. 6A). Veins dark brown, basally paler. Pterostigmatic area cloudy, with mostly simple cross veins. Costal brace dark brown. Hind wings with short triangular costal projection. Coloration of legs as in male imago.

***Abdomen*.** Coloration pattern of abdominal terga and sterna as in male imago (Fig. 6D–F). Subgenital plate apically narrowed, posterior margin rounded or slightly concave (Fig. 6G–I). Subanal plate with shallow U-shaped median emargination.

##### Description of eggs.

Oval shaped, dimensions approximately 188 × 101 μm (average values from 6 eggs). Chorionic surface slightly granulated (Fig. 6K), without distinct structures. One or two visible micropyle, shallow and rounded, located in subequatorial position (~ 12.5 μm in width) (Fig. 6J, K).

##### Main morphological diagnostics of larva.

i) abdominal sterna with more or less defined triangular maculae (Fig. 3K–N), ii) coloration of abdominal terga as on Fig. 3F, G, iii) femora with median spot (Fig. 3H, I), iv) gill plates VII narrow (in natural position from ventral view; Figs 3J, 4J), v) tergum X with short posterolateral projections (Fig. 4L, arrow), vi) abdominal terga and dorsal surface of femora with rounded spatulate setae (Fig. 4D–F); denticles along posterior margin of abdominal terga dense, irregular and pointed (Fig. 4F).

##### Main morphological diagnostics of imago.

i) abdominal sterna with narrow triangular maculae (Figs 5F, 6F), ii) coloration of abdominal terga as on Figs 5D, E, 6D, E, iii) femora with median spot (Fig. 5G), iv) wing membrane colourless (Figs 5A, 6A) (area of bullae sometimes darkened in female), v) subgenital plate of female rounded or slightly concave (Fig. 6G–I), vi) subanal plate with shallow median emargination (Fig. 6H, I), vii) penis lobes not apically widened, with shallow medio-apical notch (Fig. 5J, arrow), viii) titillators well developed, apically serrated, reaching to 0.30–0.44 of penis lobes in length (Fig. 5H, J).

##### Morphological affinities.

**Figure 7.**
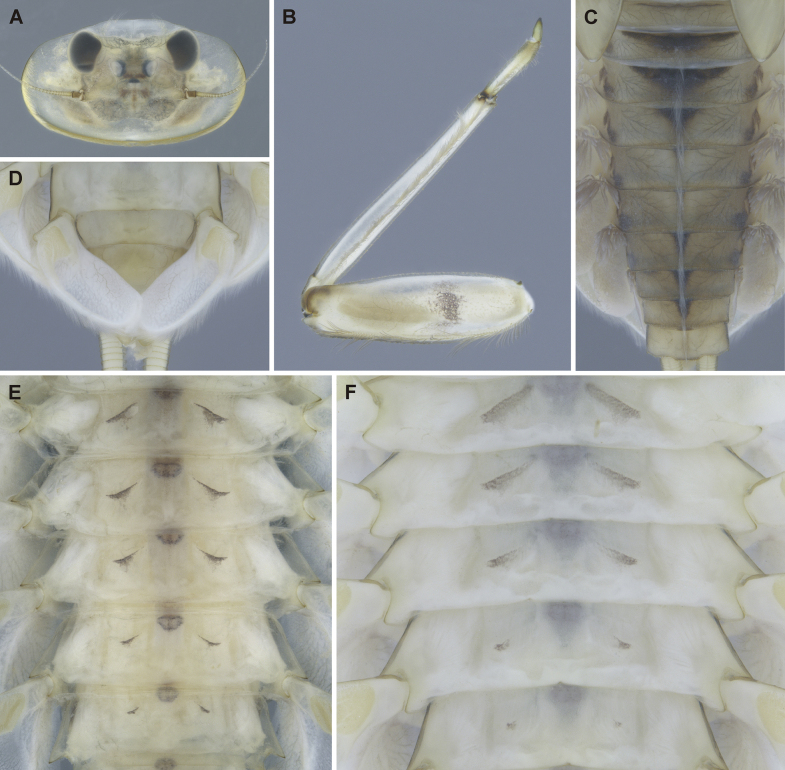
Epeorus (Caucasiron) lanceolatus sp. nov., larva **A** head of female in dorsal view (holotype) **B** fore leg in dorsal view **C** coloration of abdominal terga **D** distal part of abdomen in ventral view **E, F** coloration of abdominal sterna II–VI (**E** holotype).

**Figure 8.**
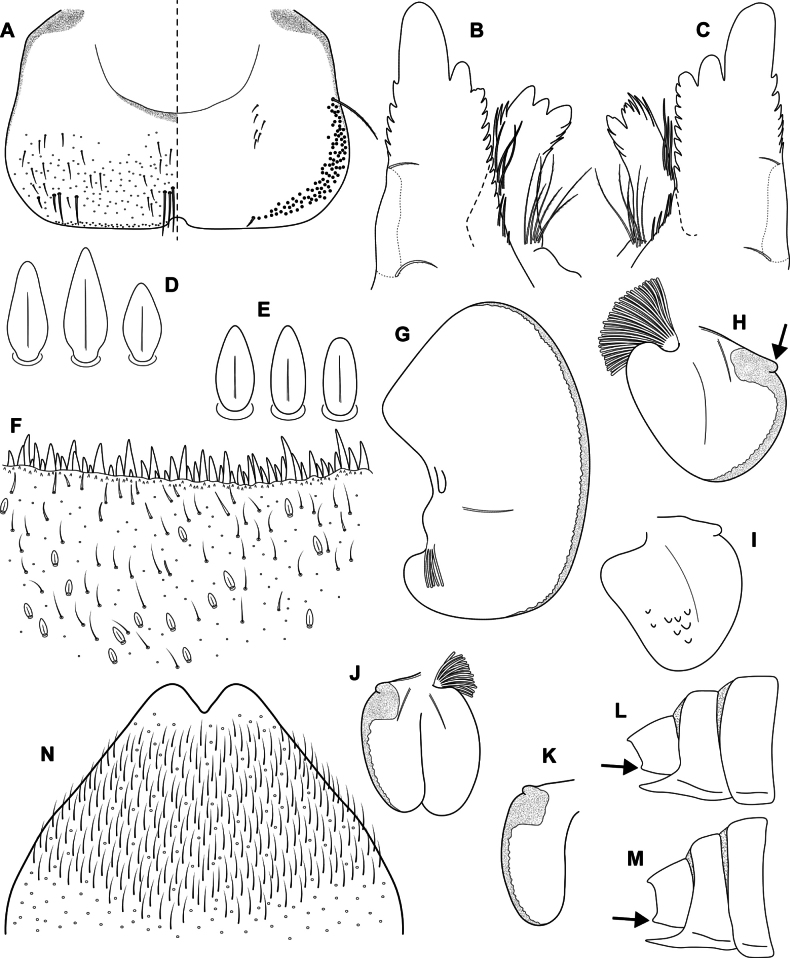
Epeorus (Caucasiron) lanceolatus sp. nov., larva **A** labrum, left half in dorsal view, right half in ventral view (black dots correspond to setae along antero-lateral margin) **B** incisors of left mandible **C** incisors of right mandible (both flattened on slide; dashed polygons correspond to area covered by setae) **D** setae on dorsal surface of femora **E** setae on surface of tergum VII **F** surface and posterior margin of abdominal tergum VII (holotype) **G** gill I **H** gill III (arrow shows projection on costal margin) **I** gill plate VI in dorsal view **J** gill VII (flattened on slide) **K** gill plate VII in ventral view **L, M** abdominal segments VIII–X in lateral view (arrow shows posterolateral projection of tergum X) **N** sternum IX of female. Drawn from late instar larva and last instar larval exuvium.

***Larva*.**Epeorus (C.) himalayensis sp. nov. is characterised by more or less defined triangular maculae on the abdominal sterna (Fig. 3K–M). This feature distinguishes E. (C.) himalayensis sp. nov. from E. (C.) guttatus, with a pair of oblique stripes and a large median macula (Fig. 13H), and E. (C.) extraordinarius, with a longitudinal reddish-brown median macula (Chen et al. 2010). The triangular maculae on abdominal sterna of E. (C.) himalayensis sp. nov. may be narrowed (Fig. 3N). Similar oblique stripes are present in E. (C.) lanceolatus sp. nov. (Fig. 7F) and E. (C.) lineatus sp. nov. (Fig. 10Q). E. (C.) himalayensis sp. nov. can be distinguished from these species based on rounded spatulate setae on abdominal terga, which are lanceolate in E. (C.) lanceolatus sp. nov. (Fig. 8E, F) and elongated spatulate in E. (C.) lineatus sp. nov. (Fig. 11E, F). In addition, E. (C.) himalayensis sp. nov. differs from the latter species by the absence of a median longitudinal line on abdominal sterna (or posteromedian macula; Fig. 10N–Q). The combination of all morphological characters that distinguish E. (C.) himalayensis sp. nov. from both related species are given in the section “Main morphological diagnostics of the larva”.

Three other species occur within the eastern part of (*E.*) *Caucasiron* range (Fig. 1), which may belong to E. (Caucasiron) based on the morphology of larvae. Of these, *E.suspicatus* possess oblique stripes on abdominal sterna (Fig. 15I). However, this species has sparse larger denticles separated by shorter denticles along the posterior margin of abdominal terga (Fig. 16F), in contrast to dense, irregular, and pointed denticles (Fig. 4F) in E. (C.) himalayensis sp. nov. Denticulation along abdominal terga separates E. (C.) himalayensis sp. nov. also from *E.psi*, with basally denticulate spines and shorter denticles (Fig. 18F). Additionally, E. (C.) himalayensis sp. nov. differs by a short dorso-apical projection on femora (Fig. 3H, I) from *E.psi* with an elongate and pointed dorso-apical projection on femora (Figs 17F, 18M).

Morphological characters separating *E.kapurkripalanorum* from *E.* (C.) *himalayensis* sp. nov. are given in the section “Remarks on *Ironparaguttatus* (Braasch, 1983) and *E.kapurkripalanorum* (Braasch, 1983)”. Considering E. (Caucasiron) species from the western part of the area, E. (C.) himalayensis sp. nov. can be easily distinguished by the shape of setae on abdominal terga. It is characterised by rounded spatulate setae, while the extralimital species have fine or basally widened hair-like setae (Hrivniak et al. 2020b).

**Figure 9.**
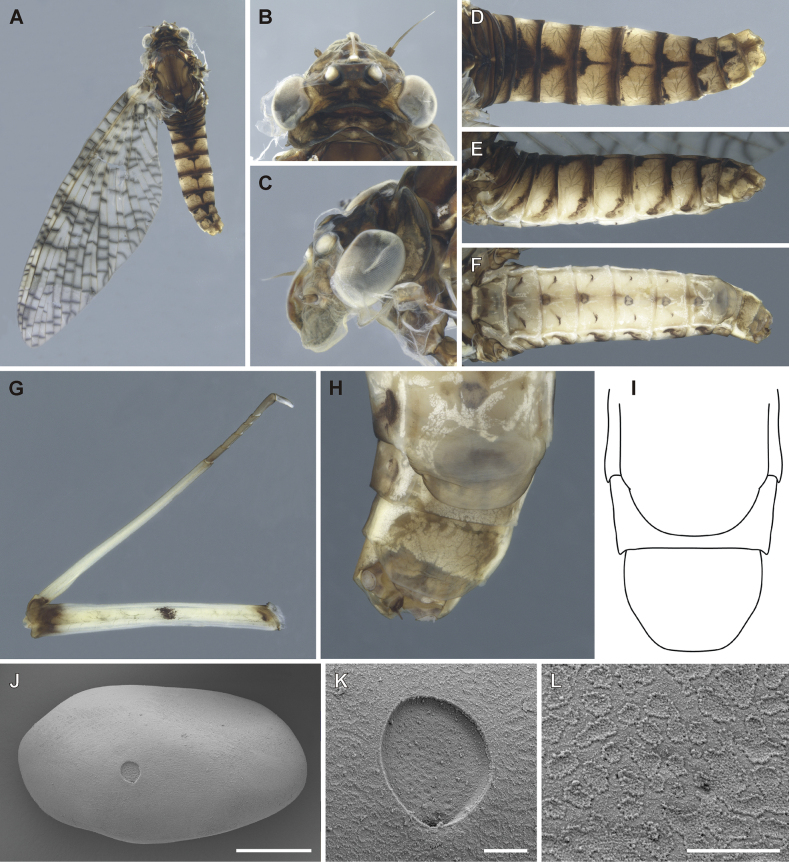
Epeorus (Caucasiron) lanceolatus sp. nov., female imago (wing and head not fully molted from subimago) **A** habitus in dorsal view **B** head in dorsal view **C** head in lateral view **D** abdomen in dorsal view **E** abdomen in lateral view **F** abdomen in ventral view **G** middle leg **H, I** subgenital and subanal plates **J** egg **K** detail of micropyle **L** texture on the surface of egg. Scale bars: 50 μm (**J**); 5 μm (**K, L**).

***Imago*.** In the eastern part of E. (Caucasiron) area, adults of four species have been described so far, namely E. (C.) guttatus (male and female), E. (C.) extraordinarius (male and female), *E.psi* (male and female) and E. (C.) lanceolatus sp. nov. (female). E. (C.) himalayensis sp. nov. can be distinguished from them by the colouration pattern of abdominal sterna, each consisting of a narrow triangular macula (Fig. 6F). This is in contrast to E. (C.) guttatus with a pair of oblique stripes and large median macula (Kluge 2015), E. (C.) extraordinarius with a longitudinal reddish-brown median macula (Chen et al. 2010) and E. (C.) lanceolatus sp. nov. with fine, slightly curved oblique stripes (Fig. 9F). From the latter species, E. (C.) himalayensis sp. nov. can be separated also by a shallow emargination on the posterior margin of subanal plate in female imago (Fig. 6G–I), which contrasts with the straight posterior margin in E. (C.) lanceolatus sp. nov. (Fig. 9H, I).

Based on male genitalia, E. (C.) himalayensis sp. nov. differs from E. (C.) guttatus by longer titillators, reaching at least 1/3 of the penis lobes (Fig. 5H, J), in contrast to short titillators not exceeding styliger in E. (C.) guttatus (Kluge 2015). The shape of penis lobes with a shallow medio-apical notch allows E. (C.) himalayensis sp. nov. to be distinguished from *E.psi*, which has apically bifurcated penis lobes with extended latero-apical tip (Eaton 1883–1888; Braasch 2006b).

Among the extralimital species, E. (C.) nigripilosus and E. (C.) caucasicus show similar coloration pattern of abdominal sterna. Epeorus (C.) himalayensis sp. nov. can be distinguished from them by relatively narrow penis lobes with a shallow medio-apical notch, because both species have apically widened penis lobes and a deeper medio-apical notch (Braasch 1979).

#### ﻿ Epeorus (Caucasiron) lanceolatus

Taxon classificationAnimaliaEphemeropteraHeptageniidae

Hrivniak & Sroka sp. nov.

98D3F3C8-D975-5D23-BBCC-E07E72D7E3F0

https://zoobank.org/4DFD1C17-701E-4BEF-9793-00F79F574355

Figs 7 , 8 , 9

##### Type material.

***Holotype***: • female larva (barcoded specimen: IN6 - labrum, leg and tergum VII mounted on slide): India Uttarakhand Pradesh, vicinity of Lambagad village, Alaknanda River, 1998 m a.s.l.; 30°38.64198'N, 79°32.02500'E (codes: IND2018/8; 40Gang); 9–11.05.2018, Martynov A.V., Palatov D. M. leg. ***Paratypes***: • 1 larva (barcoded specimen: IN2 - labrum, mandibular incisors and tergum VII mounted on slide), 1 female imago (reared from larva; barcoded specimen: L38 - larval exuvium mounted on slide): same data as holotype.

##### Etymology.

The species name *lanceolatus* (Latin) refers to lanceolate setae on abdominal terga and dorsal surface of femora characteristic for larvae.

##### Description of larva.

General coloration yellowish brown with dark brown to blackish maculation (Fig. 7). BL of late-instar larva 13.87 mm (female; *n* = 1); male unknown. Length of cerci unknown.

***Head*.** Shape oval to trapezoidal (Fig. 7A). Head dimensions of late-instar larva: length 3.5 mm, width 5.7 mm (female; *n* = 1), male unknown. Head width/length ratio: 1.53 (female; *n* = 1), male unknown. Coloration pattern of dorsal surface consists of: i) paired stripe-like and rounded maculae along epicranial suture, ii) pair of triangular (or blurred) maculae near inner edges of eyes, iii) pair of rounded maculae ventrally to lateral ocelli, iv) pale stripes extending from lateral ocelli to lateral edges of head, v) blurred or rectangular maculae between ocelli, vi) scattered maculae ventrally to median ocellus. Antennae yellowish brown, scapus and pedicellus darkened. Dorsal surface of head densely covered with elongated lanceolate setae (as on abdominal terga; Fig. 8E, F), fine hair-like setae and stick-like setae. Sparse longer and fine hair-like setae located posteriorly to eyes.

***Mouthparts*.** Labrum (Fig. 8A) widened anteriorly; anterior margin slightly rounded or straight. Lateral angles rounded. Dorsal surface sparsely covered with setae of different size, five or six longer bristle-like setae located antero-medially, and two bristles antero-laterally (Fig. 8A, left half). Epipharynx with longer, slightly plumose bristles situated along lateral to anterior margin, cluster of fine, hair-like setae medially (not figured), and group of 4–6 setae of various size (Fig. 8A, right half). Outer incisors of both mandibles with three apical teeth; outer tooth blunt in both mandibles. Inner incisor of left mandible with three apical teeth (Fig. 8B), right inner incisor bifurcated (Fig. 8C).

***Thorax*.** Prothorax anteriorly narrowed, lateral edges slightly rounded. Metanotum with small blunt posteromedian projection. Dorsal surface covered with hair-like setae, stick-like setae, and lanceolate setae (as on abdominal terga and head, Fig. 8E, F); sparse longer, hair-like setae along pro-, meso- and metanotal suture.

***Legs*.** Coloration on Fig. 7B. Femora with medial hypodermal spot, often transversely extended. Base and apex of femora darkened; patella-tibial suture darkened; tarsi proximally and distally darkened. Dorsal surface of femora covered by lanceolate setae, hair-like setae, and sparsely distributed stick-like setae (Fig. 8E; drawn from late-instar larvae and last instar larval exuvium). Dorsal edge of femora with blade-like setae. Dorsal margin of tibiae and tarsi with row of dense hair-like setae; ventral margin with irregular row of distally accumulated spines. Tarsal claws with 3–4 denticles.

***Abdominal terga*.** Colour pattern of abdominal terga consists of transversal stripe along anterior margin of terga I–IX (X) medially extending to: i) triangular macula on terga II–IV, ii) T-shaped macula on terga V–VI (VII), and iii) triangular macula on terga (VII) VIII–IX (Fig. 7C). Pair of short stripes sometimes present antero-laterally to median macula. Lateral margins with oblique stripe-like maculae on terga I–IX. Denticles along posterior margin on terga dense, relatively narrow, irregular, and pointed (Fig. 8F). Surface of terga covered with hair-like setae, stick-like setae, and lanceolate (sporadically narrow spatulate) setae (Fig. 8E, F; drawn from late-instar larvae and last instar larval exuvium). Tergum X with well-developed posterolateral projections (Fig. 8L, M, arrow). Terga with longitudinal median row of hair-like setae. Tergal spines not observed in late-instar larvae and larval exuvium from reared adult.

***Abdominal sterna*.** Yellowish, with fine oblique stripes (slightly curved in late-instar larvae; Fig. 7E, F). Nerve ganglia darkened. Sternum IX of female with V-shaped median emargination and numerous hair-like setae (Fig. 8N).

***Gills*.** Dorsal surface of gill plate I yellowish; of gill plates II–VII brownish on anterior half, greyish to brownish on posterior half. Ventral margin of all gill plates yellowish. Projection of gill plate III well developed (Fig. 8H, arrow). Gill plate VII narrow (in natural position of ventral view, Figs 7D, 8K). Dorsal margin of gill plates IV–VII with more or less developed papillae; best expressed on gill plates VI and VII (Fig. 8I).

***Cerci*.** Yellowish brown, basally darkened.

##### Description of female imago.

General coloration yellowish brown with dark brown to blackish maculation (Fig. 9A–F). Body length 14.0 mm (*n* = 1); length of cerci unknown. Length of fore wings 17.5 mm, length of hind wings unknown (broken).

***Head*.** Frons brownish; frontal fold dark brown (Fig. 9B, C). Antennae yellowish brown; scapus and pedicellus darkened. Eyes greyish, ocelli basally blackish, apically whitish.

***Thorax*.** Prothorax dark brown. Mesothorax yellowish brown; median longitudinal suture darkened. Metathorax with short posterior-median blunt projection. Furcasternum dark brown. Wing membrane of fore wings in subimago cloudy, cross veins darkened (Fig. 9A); hind wings unknown. Femora apically and basally darkened; median spot present (Fig. 9G). Tibiae apically and basally darkened, tarsi brownish. One claw blunt, one claw pointed.

***Abdomen*.** Coloration pattern of abdominal terga similar as in late-instar larvae (Fig. 9 D–F). Tergum X with medial macula. Lateral margins with oblique stripe-like maculae on terga I–IX extending dorso-posteriorly, forming transversal stripe-like macula along posterior margin of terga (Fig. 9D, E). Abdominal sterna with fine, slightly curved oblique stripes (Fig. 9F). Nerve ganglia darkened. Subgenital plate posteriorly narrowed, posterior margin slightly rounded. Subanal plate posteriorly narrowed; posterior margin straight (Fig. 9I).

***Cerci*.** Unknown.

##### Description of eggs.

Oval shaped, dimensions approximately 186 × 110 μm (average values from 7 eggs). Chorionic surface with texture as on Fig. 9L. One to three visible micropyle shallow and rounded, located in subequatorial position (~ 12.5 μm in width) (Fig. 9J, K).

**Male imago.** Unknown.

##### Main morphological diagnostics of larva.

i) abdominal sterna with fine oblique stripes (slightly curved in late-instar larvae; Fig. 7E, F), ii) coloration of abdominal terga as on Fig. 7C, iii) femora with femur spot (Fig. 7B), iv) dorsal surface of femora with lanceolate setae (Fig. 8D), v) abdominal terga with lanceolate (sporadically narrow spatulate) setae (Fig. 8E, F), vi) tergum X with well-developed posterolateral projections (Fig. 8L, M, arrow), vii) gill plates VII narrow (in natural position from ventral view; Figs 7D, 8K), viii) denticles along posterior margin of abdominal terga dense, relatively narrow, irregular and pointed (Fig. 8F).

##### Main morphological diagnostics of imago

**(female).** i) abdominal sterna with fine, slightly curved oblique stripes (Fig. 9F), ii) femora with a median spot (Fig. 9G), iii) wing membrane colourless, iv) subgenital plate posteriorly slightly rounded, iv) subanal plate posteriorly straight (Fig. 9H, I).

##### Morphological affinities.

***Larva*.**Epeorus (C.) lanceolatus sp. nov. is characterised by lanceolate setae on the dorsal surface of femora and abdominal terga (Fig. 8D, E). This trait, together with the remaining seven traits, given above distinguishes the species from all E. (Caucasiron) species described so far and also from *E.suspicatus*, whose attribution to the subgenus Caucasiron is uncertain (Figs 15, 16). Similar lanceolate setae on the dorsal surface of femora are present in *E.psi* (Fig. 18D). Epeorus (C.) lanceolatus sp. nov. differs from this species by dense, relatively narrow, irregular, and pointed denticles along the posterior margin of abdominal terga (Fig. 8F) and by a short dorso-apical projection of femora (Fig. 7B), in contrast to *E.psi* with basally denticulate spines and shorter denticles along abdominal terga (Fig. 18F) and an elongated pointed dorso-apical projection of femora (Figs 17F, 18M). The characters distinguishing *E.kapurkripalanorum* from E. (C.) lanceolatus sp. nov. are given in the section “Remarks on *Ironparaguttatus* (Braasch, 1983) and *E.kapurkripalanorum* (Braasch, 1983)”.

***Female imago*.** The presence of the fine, slightly curved oblique stripes on abdominal sterna (Fig. 9F) separates *E.* (C.) *lanceolatus* sp. nov. from E. (C.) guttatus, with a pair of oblique stripes and a large median macula on abdominal sterna (Kluge 2015), E. (C.) extraordinarius, with an longitudinal reddish brown median macula on abdominal sterna (Chen et al. 2010), E. (C.) himalayensis sp. nov., with narrow triangular maculae (Fig. 6F) and *E.psi*, with a fine longitudinal median line (sometimes reduced anteriorly) and a pair of oblique stripes on abdominal sterna (Eaton 1883–1888). In addition, E. (C.) lanceolatus sp. nov. can be distinguished from E. (C.) himalayensis sp. nov. by the straight posterior margin of subanal plate (Fig. 9H, I), in contrast to E. (C.) himalayensis sp. nov. with a shallow emargination (Fig. 6G–I).

Among the extralimital species, E. (C.) caucasicus and E. (C.) nigripilosus show similar coloration pattern on abdominal sterna (Braasch 1979). However, the female imagoes of both species have not been described, thus female genitalia cannot be compared with E. (C.) lanceolatus sp. nov. Epeorus (C.) lanceolatus sp. nov. can be separated by fine and slightly curved oblique stripes on abdominal sterna (Fig. 9F), in contrast to E. (C.) caucasicus and E. (C.) nigripilosus with well pigmented pattern (Braasch 1979). Moreover, both species are geographically restricted to the western part of the E. (Caucasiron) range (Hrivniak et al. 2020b).

#### ﻿ Epeorus (Caucasiron) lineatus

Taxon classificationAnimaliaEphemeropteraHeptageniidae

Hrivniak & Sroka sp. nov.

FC5293A0-C6C8-57C3-A1CD-A74F77CA2AF9

https://zoobank.org/6698815A-1AEE-4174-9573-91B5747B3DD8

Figs 10 , 11

##### Type material.

***Holotype***: • female larva: India: Uttarakhand Pradesh, vicinity of Badrinath town, Rishi Ganga River, right tributary of Alaknanda River, 3141 m a.s.l.; 30°44.44800'N, 79°29.34600'E; (codes: IND2018/9; 41Gang); 12–13.05.2018, Martynov A.V., Palatov D.M. leg. ***Paratypes***: • 27 larvae (barcoded specimens: L39, SP31, SP32; three larvae mounted on slide): same data as holotype. • 5 larvae (barcoded specimens: IN5 - mounted on slide, SP21, SP23): India: Uttarakhand Pradesh, vicinity of Lambagad village, Alaknanda River, 1998 m a.s.l.; 30°38.64198'N, 79°32.02500'E (codes: IND2018/8, 40Gang); 9–11.05.2018, Martynov A.V., Palatov D.M. leg. • 1 larva (barcoded specimen: GU2 - mounted on slide): Tajikistan: Gorno-Badakhshan Autonomous Region, Roshtqal’a District, left tributary of Badamdara River, 3070 m a.s.l.; 37°07.70617N’, 071°50.52000'E (code: 252Tj); 30.06.2016, Palatov D.M. leg.

##### Etymology.

The species name *lineatus* (Latin) refers to a median line on abdominal sterna characteristic for larvae.

##### Description of larva.

**Figure 10.**
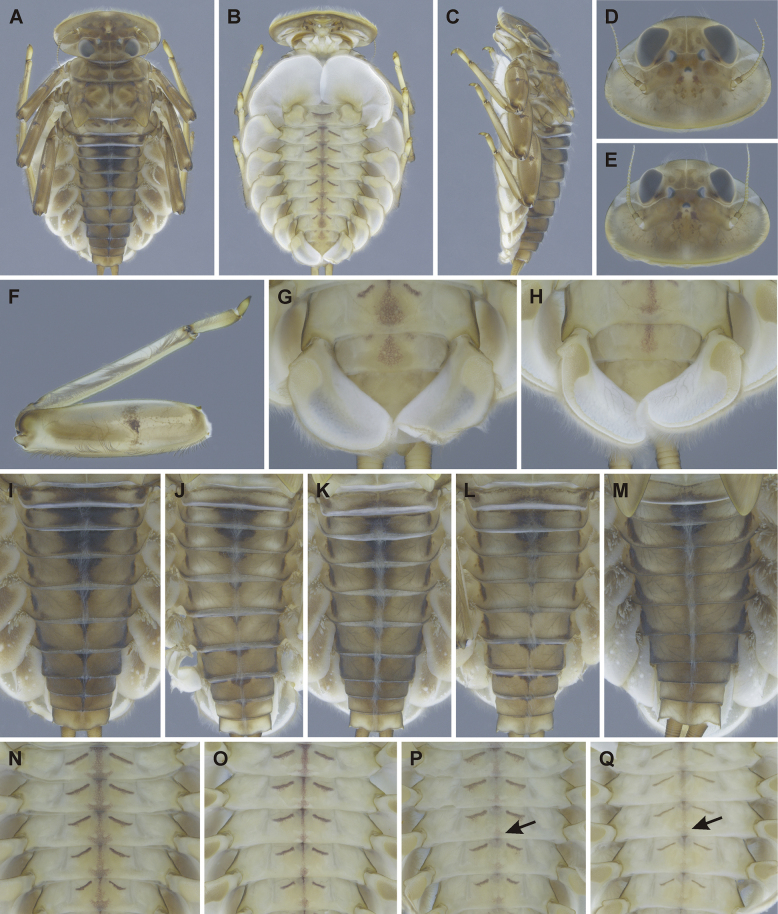
Epeorus (Caucasiron) lineatus sp. nov., larva **A** habitus in dorsal view **B** habitus in ventral view **C** habitus in lateral view **D** head of male in dorsal view **E** head of female in dorsal view **F** middle leg in dorsal view **G, H** distal part of abdomen in ventral view **I–M** coloration of abdominal terga **N–Q** coloration of abdominal sterna II–VI.

General coloration yellowish brown with dark brown to blackish maculation (Fig. 10). Body length of late-instar larva unknown. Maximum body length of examined larvae 14.0 mm (female), 9.30 mm (male). Length of cerci ~ 1.2 × body length.

**Figure 11.**
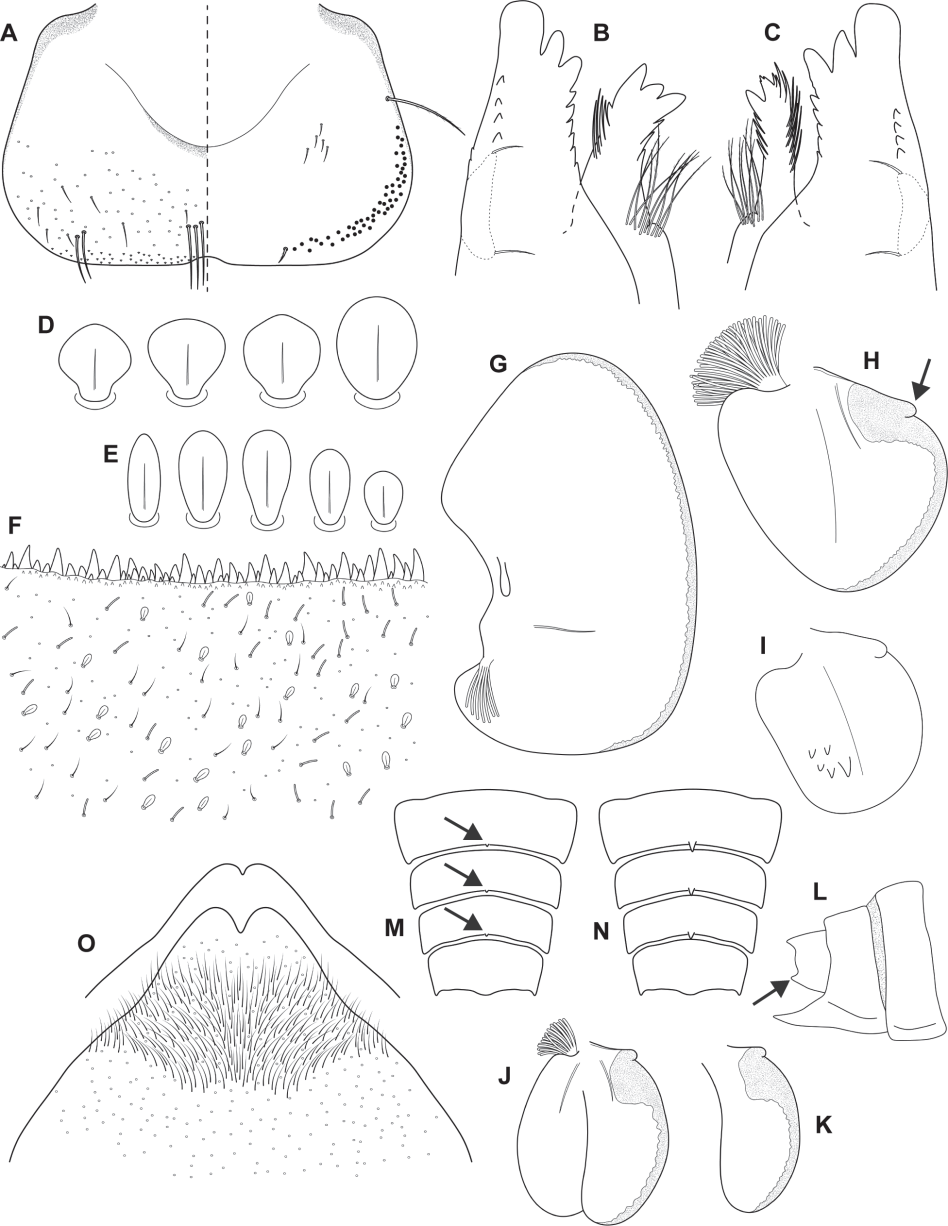
Epeorus (Caucasiron) lineatus sp. nov., larva **A** labrum, left half in dorsal view, right half in ventral view (black dots correspond to setae along antero-lateral margin) **B** incisors of left mandible **C** incisors of right mandible (both flattened on slide; dashed polygons correspond to area covered by setae) **D** setae on dorsal surface of femora **E** setae on surface of tergum VII (drawn from late-instar larva) **F** surface and posterior margin of abdominal tergum VII **G** gill I **H** gill III (arrow shows projection on costal margin) **I** gill plate VI in dorsal view **J** gill plate VII (flattened on slide) **K** gill plate VII in ventral view **L** abdominal segments VIII–X in lateral view (arrow shows posterolateral projection of tergum X) **M** abdominal terga VIII–X with short spines (arrows) **N** abdominal terga VIII–X with well-developed spines **O** sternum IX of female. Drawn from late instar larvae.

***Head*.** Shape trapezoidal (Fig. 10D, E). Head width/length ratio: 1.49–1.56 (female; *n* = 6), 1.50–1.57 (male; *n* = 2). Coloration pattern of dorsal surface consists of: i) paired stripe-like and rounded maculae along epicranial suture, ii) pair of triangular (or blurred) maculae near inner edges of eyes, iii) pair of rounded maculae ventrally to lateral ocelli, iv) pale stripes extending from lateral ocelli to lateral edges of head, v) rectangular or blurred macula between ocelli, vi) scattered smaller maculae ventrally to median ocellus. Antennae yellowish brown, scapus and pedicellus darkened. Dorsal surface covered with elongated spatulate setae (as on abdominal terga; Fig. 11E), fine hair-like setae and stick-like setae. Sparse longer and fine hair-like setae located posteriorly to eyes.

***Mouthparts*.** Labrum (Fig. 11A) widened anteriorly; anterior margin slightly rounded or nearly straight. Lateral angles rounded. Dorsal surface sparsely covered with setae of different size, 5–6 longer bristle-like setae located antero-medially, and two bristles antero-laterally (Fig. 11A, left half). Epipharynx with longer, slightly plumose bristles situated along lateral to anterior margin, cluster of fine hair-like setae medially (not figured), and 5–9 setae of various size (Fig. 11A, right half). Outer incisors of both mandibles with three apical teeth; outer tooth blunt in both mandibles. Inner incisor of left mandible with three apical teeth (Fig. 11B), right inner incisor bifurcated (Fig. 11C).

***Thorax*.** Prothorax anteriorly narrowed, lateral edges slightly rounded. Metanotum with small blunt posteromedian projection. Dorsal surface covered with hair-like setae, stick-like setae and elongated spatulate setae (as on abdominal terga and head); sparse longer, hair-like setae along pro-, meso-, and metanotal suture.

***Legs*.** Coloration as on Fig. 10F. Femora with medial hypodermal spot, often transversely extended. Base and apex of femora darkened; patella-tibial suture darkened; tarsi proximally and distally darkened. Dorsal surface of femora covered by rounded (sporadically apically narrowed) spatulate setae (Fig. 11D), hair-like setae, and sparsely distributed stick-like setae. Dorsal edge of femora with blade-like setae. Dorsal margin of tibiae and tarsi with row of dense hair-like setae; ventral margin with irregular row of distally accumulated spines. Tarsal claws with 2–3 denticles.

***Abdominal terga*.** Colour pattern of abdominal terga consists of transversal stripe along anterior margin of terga I–IX (X) medially extending to: i) triangular, rounded, or anteriorly and posteriorly widened macula on terga II–IV; and ii) triangular or T-shaped macula on terga V–IX (Fig. 10I–M). Lateral margins with oblique stripe-like maculae on terga I–IX, sometimes dorso-posteriorly extended. Denticles along posterior margin on terga dense, irregular, and pointed (Fig. 11F). Surface of terga covered with hair-like setae, stick-like setae, and elongated (sporadically rounded) spatulate setae (Fig. 11E, F) (dominantly rounded spatulate setae can be present in younger instars). Tergum X with well-developed posterolateral projections (Fig. 11L, arrow). Terga with longitudinal median row of hair-like setae. More or less developed posteromedian spine (most expressed on terga VII–IX (Fig. 11M, N). Posteromedian tergal spine observed only in larvae of BL 4.6–11.2 mm (*n* = 22; barcoded specimens SP21, SP31, L39, SP23, IN5), not observed in larger larvae of BL 11.2–14.0 (*n* = 5; barcoded specimens: GU2, SP32).

***Abdominal sterna*.** Yellowish, with a pattern consisting of oblique stripes and median line extending from anterior to posterior margin (Fig. 10N–Q). Median line often posteriorly widened (Fig. 11N) or reduced to posteromedian macula Fig. 11P, Q, arrows). Sternum IX of female apically narrowed, with V-shaped median emargination, and numerous hair-like setae (Fig. 11O).

***Gills*.** Dorsal surface of gill plate I yellowish; of gill plates II–VII brownish. Ventral margin of all gill plates yellowish. Projection of gill plate III well-developed (Fig. 11H, arrow). Gill plate VII wide (in natural position of ventral view, Figs 10G, H, 11K). Dorsal margin of gill plates (III) IV–VII with more or less developed papillae; best expressed on gill plates VI and VII (Fig. 11I).

***Cerci*.** Yellowish brown, basally darkened.

##### Imagoes and eggs.

Unknown.

##### Main morphological diagnostics of the larva.

i) abdominal sterna with oblique stripes and more or less developed median line (Fig. 10N–Q), ii) coloration of abdominal terga as on Fig. 10I–M, iii) femora with median spot (Fig. 10F), iv) gill plates VII wide (in natural position from ventral view; Figs 10G, H, 11K), v) tergum X with well-developed posterolateral projections (Fig. 11L, arrow), vi) abdominal terga with elongated (sporadically rounded) spatulate setae (Fig. 11E, F), vii) dorsal surface of femora with rounded spatulate setae (Fig. 11D), viii) denticles along posterior margin on terga dense, irregular and pointed (Fig. 11F).

##### Morphological affinities.

***Larva*.** Based on the coloration pattern of abdominal sterna consisting of oblique stripes and more or less developed longitudinal median line, E. (C.) lineatus sp. nov. can be distinguished from E. (C.) extraordinarius, with a longitudinal reddish brown median macula (Chen et al. 2010) and E. (C.) himalayensis sp. nov., with more or less defined triangular maculae (Fig. 3K–N). In addition, E. (C.) lineatus sp. nov. differs from the latter species by elongated spatulate setae on abdominal terga (Fig. 11E, F) and wide shape of gill plates VII (Figs 10G, H, 11K), in contrast to rounded spatulate setae and narrow gill plates VII in E. (C.) himalayensis sp. nov. (Figs 3J, 4J).

Elongated spatulate setae on abdominal terga separate E. (C.) lineatus sp. nov. from E. (C.) lanceolatus sp. nov., with lanceolate setae on abdominal terga (Fig. 8E). Other characters distinguishing E. (C.) lineatus sp. nov. from E. (C.) himalayensis sp. nov. and E. (C.) lanceolatus sp. nov. are given in the section “Main morphological diagnostics of the larva”.

Epeorus (C.) lineatus sp. nov. is most similar to Central Asian E. (C.) guttatus. Both species possess elongated spatulate setae on the dorsal margin of abdominal terga, well-developed posterolateral projection on tergum X, wide gill plates VII and similar coloration of abdominal terga and legs. Epeorus (C.) lineatus sp. nov. can be distinguished by the presence of more or less developed longitudinal median line on abdominal sterna (Fig. 10N–Q), in contrast to E. (C.) guttatus with large median macula (Fig. 13H). A longitudinal median line in E. (C.) lineatus sp. nov. is sometimes posteriorly widened (Fig. 10N). When it is pronounced, reaching oblique stripes, the pattern may resemble that of E. (C.) guttatus. This was observed in a specimen collected in Tajikistan (barcoded specimen: GU2).

Epeorus (C.) lineatus sp. nov. differs from other Epeorus species, which could represent the subgenus Caucasiron based on larval morphology (Fig. 1), by dense, irregular, and pointed denticles along posterior margin of abdominal terga (Fig. 11F). *Epeorussuspicatus* possesses sparse larger denticles separated by shorter denticles (Fig. 16F) and *E.psi* basally denticulate spines and short denticles (Fig. 18F). The latter species additionally differs by a long and pointed dorso-apical projection of femora (Figs 17F, 18M). Morphological diagnostics of *E.kapurkripalanorum* are given in the section “Remarks on *Ironparaguttatus* (Braasch, 1983) and *E.kapurkripalanorum* (Braasch, 1983)”.

Epeorus (C.) lineatus sp. nov. differs from all extralimital species of the subgenus Caucasiron by the presence of spatulate setae on abdominal terga, in contrast to fine or basally widened hair-like setae present in E. (Caucasiron) from the western part of its range (Hrivniak et al. 2020b).

### ﻿Distribution and habitat of the new Epeorus (Caucasiron) species

**Figure 12.**
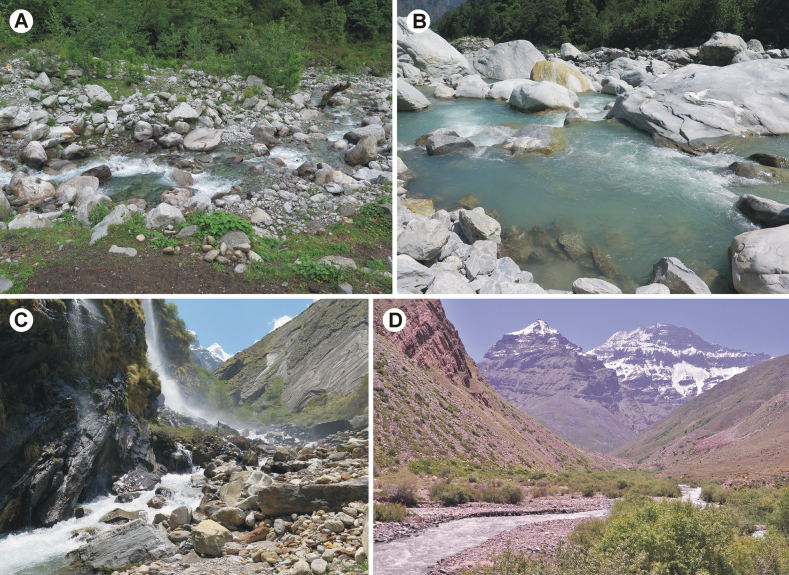
Streams and rivers inhabited by Epeorus (Caucasiron) spp. in the Himalayas (**A–C**) and the Pamir Mountains (**D**) **A** the type locality of E. (C.) himalayensis sp. nov. **B** locality of E. (C.) himalayensis sp. nov., E. (C.) lineatus sp. nov. and E. (C.) lanceolatus sp. nov. (type locality) **C, D** localities of E. (C.) lineatus sp. nov. (**C** type locality).

Epeorus (C.) himalayensis sp. nov. and E. (C.) lanceolatus sp. nov. are currently known only from the western part of the Himalayas in north-west India (Fig. 2). Epeorus (C.) lineatus sp. nov. was recorded in the Himalayas (north-west India) and in the Pamir Mountains in Central Asia (Tajikistan; Fig. 2). All species inhabit mountain streams and rivers with stony bed substrate and predominant riffles with turbulent flow. Epeorus (C.) himalayensis sp. nov. was found between 1998 and 2099 m a. s. l. (Fig. 12A, B), while E. (C.) lineatus sp. nov. occurred in India at altitudes of 1998 and 3141 m a. s. l. and in Tajikistan at 3070 m a. s. l. (Fig. 12B–D). Epeorus (C.) lanceolatus sp. nov. was only found in one river at an altitude of 1998 m a. s. l. (Fig. 12B) and was absent from the smaller streams investigated in the area. Adults of E. (C.) himalayensis sp. nov. and E. (C.) lanceolatus sp. nov. were reared in the field in May.

### ﻿Morphological revisions

In this section, we provide the list of main diagnostic characters, drawings, and photographs of previously described species attributable to E. (Caucasiron) from our study area (Central Asian mountains and the Himalayas). The subgeneric attribution is regarded as uncertain for *E.suspicatus* and *E.psi* (see below). The purpose of this chapter is to facilitate the direct comparison of all potential E. (Caucasiron) species from our study area based on the same set of characters.

#### ﻿ Epeorus (Caucasiron) guttatus

Taxon classificationAnimaliaEphemeropteraHeptageniidae

(Braasch & Soldán, 1979)

BBD01E04-00F0-5F0F-AAC5-7955AC81217F

Figs 13 , 14

Iron guttatus Braasch & Soldán, 1979.
Epeorus (Iron) guttatus (Braasch & Soldán, 1979): Kluge 1988: 296.
Epeorus (Caucasiron) guttatus (Braasch & Soldán, 1979): Kluge 1997: 234.
Iron (Caucasiron) guttatus (Braasch & Soldán, 1979): Braasch 2006b: 87.

##### Type locality.

Kazakhstan: Issyk River near Alma-Ata.

##### Examined material

**(deposited in IECA).** • 1 larva: paratype from type locality. • 1 larva (barcoded specimen: GU3): Kyrgyzstan: Chuy Region, left tributary of Ala-Archa River, 1717 m a. s. l., 42°37.743'N, 74°29.293'E (code: 1Kyrgyz), 26. 05. 2016, Palatov D.M. leg. • 6 larvae (barcoded specimen: GU71K): Kyrgyzstan: Osh Region, right tributary of Kulun River, 2060 m a. s. l., 40°29.516'N, 74°09.077'E (code: 71Kyrgyz), 01. 05. 2017, Palatov D.M. leg. • 3 larvae (barcoded specimen: GU1): Kyrgyzstan: Osh Region Kara-Bel River, 2135 m. a. s. l., 40°30.300'N, 74°10.621'E (code: 73Kyrgyz), 01. 05. 2017, Palatov D. M. leg.

##### Distribution, habitat, and biology.

Tian Shan: Kazakhstan (Braasch and Soldán 1979; Kluge 2015), Kyrgyzstan; Pamir: Tajikistan (Kluge 2015) (Fig. 1). The species inhabits mountain streams with rapid turbulent flow and bed substrate formed by stones and boulders (Kluge 2015). The altitude of our sampling sites ranged between 1717 and 2135 m a.s.l. Adults were recorded from May to September (Kluge 2015).

##### Main morphological diagnostics of the larva.

**Figure 13.**
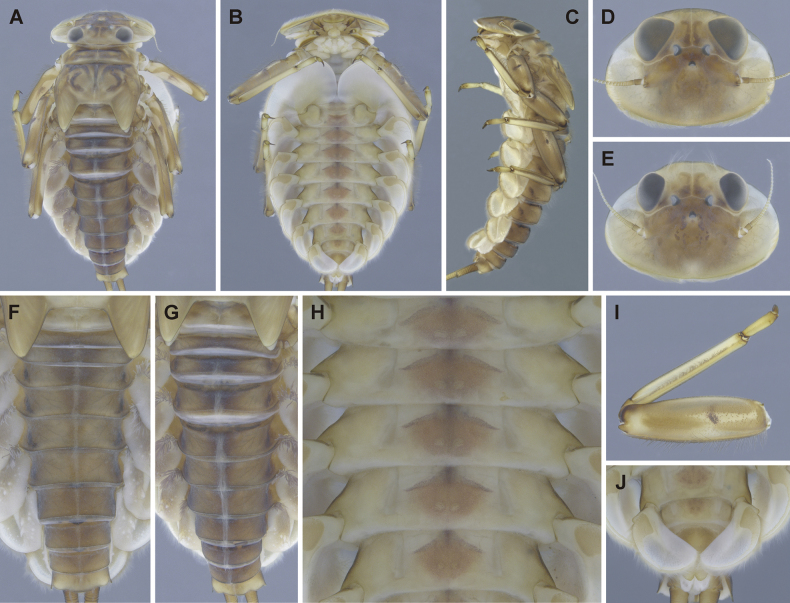
Epeorus (Caucasiron) guttatus, larva **A** habitus in dorsal view **B** habitus in ventral view **C** habitus in lateral view **D** head of male in dorsal view **E** head of female in dorsal view **F, G** coloration of abdominal terga **H** coloration of abdominal sterna II–VI **I** middle leg in dorsal view **J** distal part of abdomen in ventral view.

**Figure 14.**
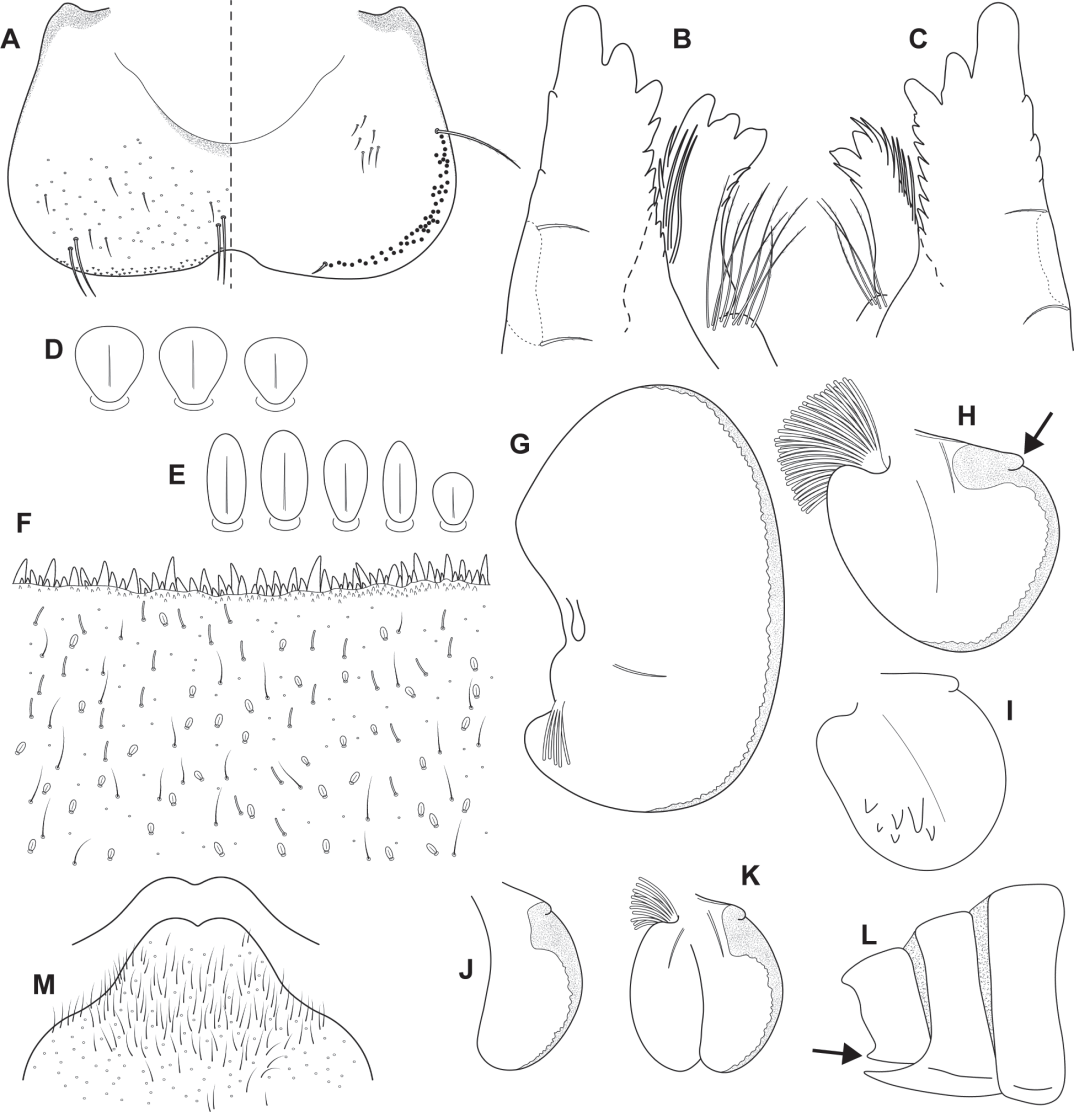
Epeorus (Caucasiron) guttatus, larva **A** labrum, left half in dorsal view, right half in ventral view (black dots correspond to setae along antero-lateral margin) **B** incisors of left mandible **C** incisors of right mandible (both flattened on slide; dashed polygons correspond to area covered by setae) **D** setae on dorsal surface of femora **E** setae on surface of tergum VII **F** surface and posterior margin of abdominal tergum VII (paratype) **G** gill I **H** gill III (arrow shows projection on costal margin) **I** gill plate VI in dorsal view **J** gill plate VII in ventral view **K** gill VII (flattened on slide) **L** abdominal segments VIII–X in lateral view (arrow shows posterolateral projection of tergum X) **M** sternum IX of female. Drawn from late instar larvae.

i) femora with a median spot (Fig. 13I), ii) abdominal sterna with a pair of oblique stripes and a large median macula (Fig. 13H), iii) coloration of abdominal terga as on Fig. 13F, G, iv) tergum X with a well-developed posterolateral projection (Fig. 14L, arrow), v) dorsal surface of femora with rounded spatulate setae (Fig. 14D), vi) abdominal terga with elongated (sporadically rounded) spatulate setae (Fig. 14E, F), vii) gill plates VII wide (in natural position from ventral view) (Figs 13J, 14J), viii) projection on gill plates III well-developed (Fig. 14H), ix) denticles along posterior margin of abdominal terga dense, irregular, and pointed (Fig. 14F).

##### Remarks.

***Morphology*.** Description of adult stages in Kluge (2015).

#### ﻿ Epeorus (Caucasiron ?) suspicatus

Taxon classificationAnimaliaEphemeropteraHeptageniidae

 (Braasch, 2006)

C44E9428-B608-507E-B0A8-8C493969FF85

Figs 15 , 16

Iron suspicatus Braasch, 2006.
Epeorus (Caucasiron) suspicatus (Braasch, 2006): Vasanth et al. 2021: 519.

##### Type locality.

Nepal: Tal, Marshyangdi River (orig. Marsyandi-Tal, Thangja) (2400 m a. s. l.).

##### Examined material

**(deposited in SMNS).** • 2 larvae: holotype and paratype from type locality. • 1 larva (paratype): Nepal: Marsyandi-Tal, Bagarchap, ca. 2100 m a. s. l., 21.05.1980, leg. Sivec.

##### Distribution, habitat, and biology.

Himalayas: Nepal and India (Fig. 1). The species inhabits rhithral zones of mountain streams. The altitude of the sampling sites ranged between 2000 and 2400 m a. s. l. Late-instar larvae were recorded in May (Braasch 2006a).

##### Main morphological diagnostics of the larva.

**Figure 15.**
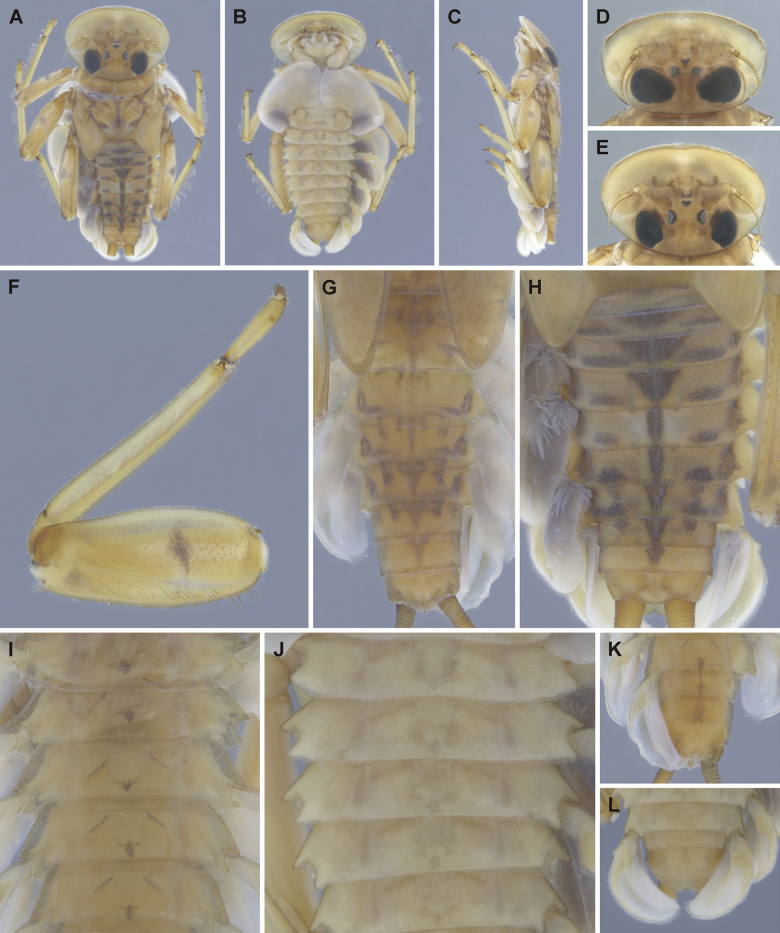
*Epeorus* (*Caucasiron*?) *suspicatus*, larva **A** habitus in dorsal view **B** habitus in ventral view **C** habitus in lateral view **D** head of male in dorsal view (holotype) **E** head of female in dorsal view **F** middle leg in dorsal view **G, H** coloration of abdominal terga (**G** holotype) **I–J** coloration of abdominal sterna II–VI (**I** holotype) **K, L** distal part of abdomen in ventral view (**K** holotype).

**Figure 16.**
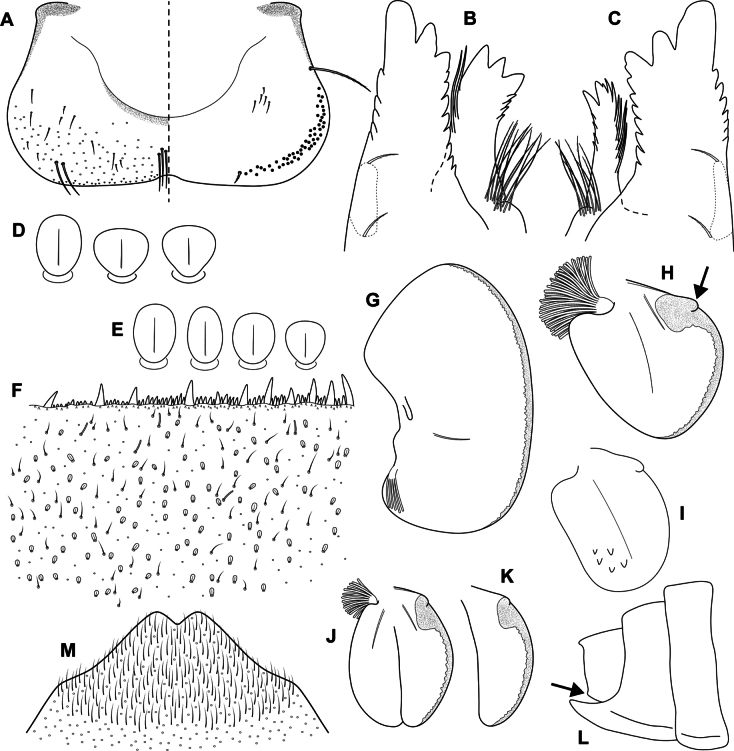
*Epeorus* (*Caucasiron*?) *suspicatus*, larva **A** labrum, left half in dorsal view, right half in ventral view (black dots correspond to setae along antero-lateral margin) **B** incisors of left mandible **C** incisors of right mandible (both flattened on slide; dashed polygons correspond to area covered by setae) **D** setae on dorsal surface of femora **E** setae on surface of tergum VII **F** surface and posterior margin of abdominal tergum VII **G** gill I **H** gill III (arrow shows projection on costal margin) **I** gill plate VI in dorsal view **J** gill plate VII (flattened on slide) **K** gill plate VII in ventral view **L** abdominal segments VIII–X in lateral view (arrow shows indistinct posterolateral projection of tergum X) **M** sternum IX of female. Drawn from late instar larvae.

i) abdominal sterna with a pair of oblique stripes and a posterio-median macula (Fig. 15I), ii) coloration of abdominal terga as on Fig. 15G, H, iii) posterior margin of abdominal terga with sparse larger denticles separated by shorter denticles (Fig. 16F), iv) tergum X with a short posterolateral projection (Fig. 16L, arrow), v) gill plates VII narrow (in natural position from ventral view) (Figs 15K, L, 16K), vi) projection on gill plates III well-developed (Fig. 16H, arrow), vii) femora with a median spot (Fig. 15F), viii) dorsal surface of femora with rounded spatulate setae (Fig. 16D), ix) abdominal terga with elongated (oval) and rounded spatulate setae (Fig. 16E, F).

##### Remarks.

***Morphology*.** Adult stages undescribed.

***Taxonomy*.** The attribution of the species to the subgenus E. (Caucasiron) by Vasanth et al. (2021) was not confirmed by male genitalia or molecular data and hence remains unclear.

#### ﻿ Epeorus (Caucasiron ?) psi

Taxon classificationAnimaliaEphemeropteraHeptageniidae

(Eaton, 1885)

8339538B-878F-522F-B270-B9D2A9E0B11F

Figs 17 , 18

Iron psi ? (*Eaton*, 1885): Braasch 1980b: 58.
Epeorus (Belovius) psi (Eaton, 1885): Tshernova 1981: 332.
Epeorus (Caucasiron) psi (Eaton, 1885): Vasanth et al. 2021: 516-519.

##### Type locality.

India: Kullu district (orig. Kooloo, Himalaya) (Eaton 1883–1888).

##### Examined material

**(deposited in IECA and NMNH NASU).** • 20 larvae (barcoded specimens: IN4, IN42 - both mounted on slide), 2 male subimagoes (barcoded specimen: IN44 - genitalia mounted on slide): India: Uttarakhand state, vicinity of Guptkashi town, Madhyamaheshwar Ganga Mandahishvar River – left tributary of Mandakini River, 30°32.27700'N, 79°05.95698'E, 1102 m a.s.l., 15.–16.5.2018, Martynov A.V. leg. (code: IND2018/11). • 5 larvae (barcoded specimen: IN43 - mounted on slide): India: Uttarakhand Pradesh, vicinity of Guptkashi village, Mandakini River, 1087 m a.s.l., 30°32.24700'N, 79°05.73900'E, 16.05.2018, Martynov A.V. leg. (code: IND 2018/12).

##### Distribution, habitat, and biology.

Himalayas: India (Eaton 1883–1888; Vasanth et al. 2021), Nepal (Braasch 1980b, 1981), south-east Tibet (Ma and Zhou 2022); Hengduan Shan and Yunnan-Guizhou Plateau: China (Ma and Zhou 2022) (Fig. 1). The species inhabits mountain streams and rivers in relatively wide altitudinal range. The altitude of the sampling sites ranged between 488 and 2100 m a.s.l. (our data; Braasch 1980b, 1981; Vasanth et al. 2021). Adults were recorded in May (Braasch 1980b).

##### Main morphological diagnostics of the larva.

**Figure 17.**
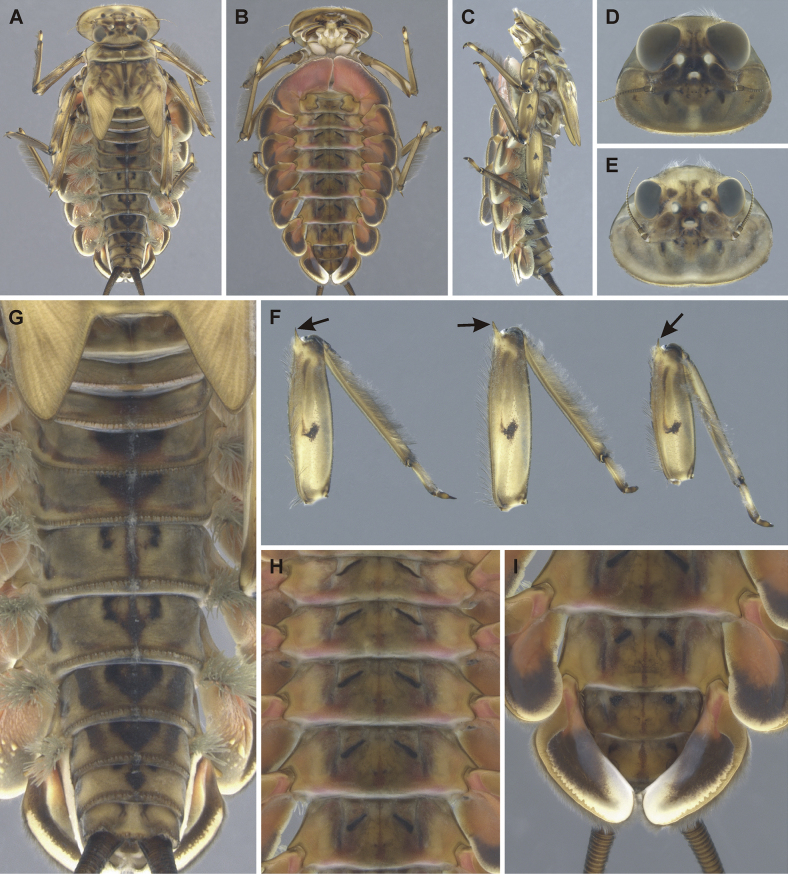
*Epeorus* (*Caucasiron*?) *psi*, larva **A** habitus in dorsal view **B** habitus in ventral view **C** habitus in lateral view **D** head of male in dorsal view **E** head of female in dorsal view **F** legs in dorsal view (arrows point to elongated and pointed dorso-apical projections) **G** coloration of abdominal terga **H** coloration of abdominal sterna II–VI **I** distal part of abdomen in ventral view.

**Figure 18.**
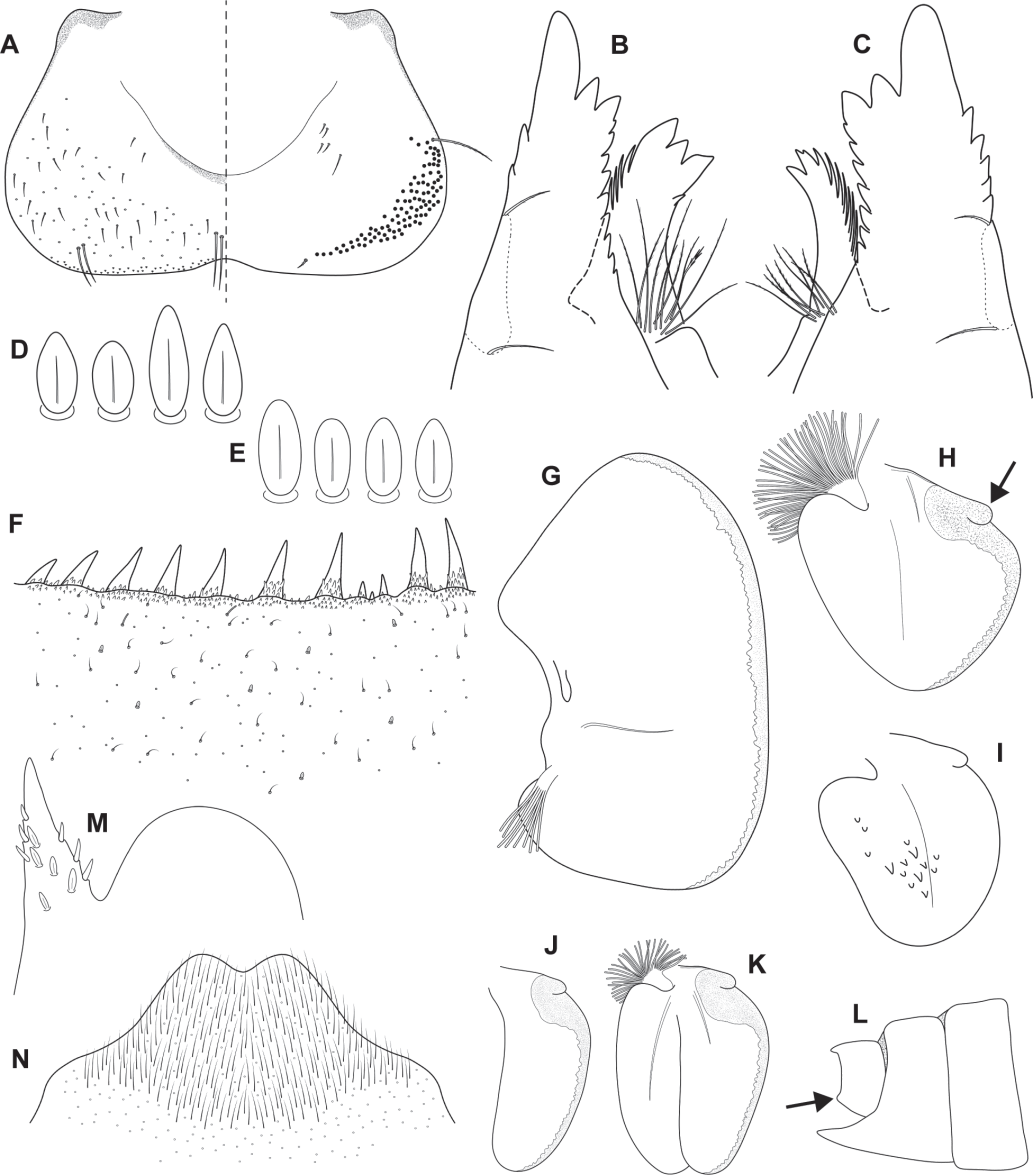
*Epeorus* (*Caucasiron*?) *psi*, larva **A** labrum, left half in dorsal view, right half in ventral view (black dots correspond to setae along antero-lateral margin) **B** incisors of left mandible **C** incisors of right mandible (both flattened on slide; dashed polygons correspond to area covered by setae) **D** setae on dorsal surface of femora **E** setae on surface of tergum VII **F** surface and posterior margin of abdominal tergum VII **G** gill I **H** gill III (arrow shows projection on costal margin) **I** gill plate VI in dorsal view **J** gill plate VII in ventral view **K** gill plate VII (flattened on slide) **L** abdominal segments VIII–X in lateral view (arrow shows posterolateral projection of tergum X) **M** detail of dorso-apical projection of femora (middle leg) **N** sternum IX of female. Drawn from late instar larvae.

i) abdominal sterna with a pair of oblique stripes and a longitudinal median line (sometimes reduced anteriorly) (Fig. 17H), ii) coloration of abdominal terga as on Fig. 17G), posterior margin of abdominal terga with basally denticulate spines and shorter denticles (Fig. 18F), iv) tergum X with a short posterolateral projection (Fig. 18L, arrow), v) gill plates VII narrow (in natural position from ventral view) (Figs 17I, 18J), vi) projection on gill plates III well-developed (Fig. 18H, arrow), vii) femora with median femur spot (Fig. 17F), viii) dorsal surface of femora with lanceolate (sporadically elongated spatulate) setae (Fig. 18D), ix) abdominal terga with elongated spatulate and lanceolate setae (Fig. 18E), femora with an extended and pointed dorso-apical projection (Figs 17F, 18M).

##### Remarks.

***Morphology*.** The species was originally described by Eaton (1883–1888) based on male and female imago. The larva and the male subimago were later described by Braasch (1980b) from Nepal. Association with imagoes of *E.psi* was based on the specific pattern of colouration on abdomen and the shape of penis lobes.

***Taxonomy*.** The species was attributed to the subgenus Iron within its own species group “*Ironpsi*-Gruppe” (Braasch 1980b, 2006b). Vasanth et al. (2021) examined the morphology of larvae and assigned the species to the subgenus Caucasiron based on “the shape of gills II–VII with an outer thumb-like projection”. Although the shape of gill plates II–VII is similar to those of E. (Caucasiron), male genitalia with bifurcated penis lobes and extended latero-apical tip, as figured by Eaton (1883–1888) and described by Braasch (1980b, 2006b), are not consistent with the diagnosis of E. (Caucasiron) as given by Kluge (1997, 2015). Therefore, the systematic position of *E.psi* remains unclear until the systematic revision based on molecular data is available.

***Distribution*.** Specimens from Taiwan identified as *E.psi* by Ulmer (1912) were later described as *E.erratus* Braasch, 1981.

### ﻿Remarks on *E.paraguttatus* (Braasch, 1983) and *E.kapurkripalanorum* (Braasch, 1983)

Two other species from our study area have been assigned to E. (Caucasiron) in the literature; however, their systematic position is rather doubtful. Braasch (1981) attributed a single larva, a male and a female imago as well as two female subimagoes from Nepal to E. (C.) guttatus. Later, Braasch (1983b) re-evaluated these specimens as a new species that he described as *Ironparaguttatus* Braasch, 1983. The gill plate III of *I.paraguttatus* larvae bears a projection on the costal margin (Braasch 1983b: 196, fig. 2), which corresponds to the diagnosis of the subgenus Epeorus (Caucasiron). Therefore, the species was classified as *Iron* (*Caucasiron*?) *paraguttatus* in Braasch (2006b). However, its subgeneric attribution was marked as uncertain (Kluge 2015), because the male genitalia of the holotype (Braasch 1981: 106, fig. 1r, s, t) possesses penis lobes with a latero-apical spine, which is not consistent with the definition of E. (Caucasiron) according to Kluge (1997, 2015). For this reason, we assume that *I.paraguttatus* does not belong to E. (Caucasiron) and was excluded from our study.

Assuming from the shape of genitalia as figured in Braasch (1981), *I.paraguttatus* can most likely be attributed to the *montanus* species group within the subgenus E. (Iron) (Brodsky 1930; Kluge 2015). Importantly, the larva assigned to *I.paraguttatus* does not belong to the type series of this species. It was collected in different locality than the adults and was associated with them only based on the similarity in the coloration pattern of the abdomen. We assume that the larva represents a different species than the adults described as *I.paraguttatus*. According to the description of Braasch (1981, 1983b), the larva is characterised by: the absence of coloration pattern on abdominal sterna, the presence of a triangular median macula on terga VI–VIII, the shape of the gill plate VII, and the shape of tarsal claws. These larval morphological characters are not consistent with any of the species described herein and it may indicate that another unnamed species of E. (Caucasiron) occurs in the Himalayas.

Another problematic species, *Epeoruskapurkripalanorum*, was originally described from the western Himalayas (Spiti Valley) as *Ironopsis* sp. 1. (Kapur and Kripalani 1963). Braasch (1983b) assigned these specimens to the name *Ironkapurkripalanorum* Braasch, 1983, relying solely on the original description and figures from Kapur and Kripalani (1963), but not on the investigation of the material. Vasanth et al. (2021) attributed this species to E. (Caucasiron) based on the presence of a projection on the costal margin of gill plates II figured in the original description (Kapur and Kripalani 1969: 213, fig. 14b, c). *Epeoruskapurkripalanorum* was not included in our molecular analyses and morphological investigation due to the lack of available material. As the adult stages are not known, its systematic position in E. (Caucasiron) remains unclear. Moreover, the larva was insufficiently described and significant morphological traits necessary for species identification, such as the coloration of abdominal sterna or the shape of setae on the surface of femora and abdominal terga, were not provided. Nevertheless, based on the original description by Kapur and Kripalani (1963) and the statements of other authors (Braasch 2006a; Vasanth et al. 2021), the larva of *E.kapurkripalanorum* differs from all species described here by the absence of a median spot on the dorsal surface of femora, the presence of a median longitudinal dense row of long hair-like setae and distinctly “over-bent” tarsal claw of fore legs (Kapur and Kripalani 1963: 215, fig. 16a, d). The median longitudinal dense row of long hair-like setae and a shallow convexity on the costal margin of gill plates II–VII are characteristic of *E.rheophilus*, which is distributed in Central Asia (Kluge 2015) and the Himalayas (unpublished data). Therefore, it cannot be ruled out that *E.kapurkripalanorum* is related to *E.rheophilus*, rather than to E. (Caucasiron).

## ﻿Conclusions

Our study confirms that the diversity of E. (Caucasiron) in the Central Asian mountains and the Himalayas is higher than previously known. This is supported by the descriptions of three new species, namely E. (C.) himalayensis sp. nov., E. (*C.*) *lanceolatus* sp. nov. and E. (C.) lineatus sp. nov., discovered in relatively restricted area in Pamir and the western part of the Himalayas. In addition to the morphological descriptions and DNA barcoding of the new species, we present the main larval diagnostic characters of the already known E. (C.) guttatus and two other species, *E.psi* and *E.suspicatus*, possibly related to E. (Caucasiron). The DNA barcodes for E. (C.) guttatus and *E.psi* are provided, which may facilitate direct comparison between the species and the discovery of greater diversity of this mayfly lineage in Central Asia and the Himalayas. The literature review of dubious species indicates that *E.paraguttatus* does not belong to E. (Caucasiron). The systematic position of *E.kapurkripalanorum* remains unclear.

## Supplementary Material

XML Treatment for Epeorus (Caucasiron) himalayensis

XML Treatment for Epeorus (Caucasiron) lanceolatus

XML Treatment for Epeorus (Caucasiron) lineatus

XML Treatment for Epeorus (Caucasiron) guttatus

XML Treatment for Epeorus (Caucasiron ?) suspicatus

XML Treatment for Epeorus (Caucasiron ?) psi

## ﻿Additional information

### Author ORCIDs

Ľuboš Hrivniak https://orcid.org/0000-0002-9327-1314

Pavel Sroka https://orcid.org/0000-0003-4367-6564

Roman J. Godunko https://orcid.org/0000-0003-2196-3327

Alexander V. Martynov https://orcid.org/0000-0002-6506-5134

Jindřiška Bojková https://orcid.org/0000-0003-0996-3308

### Data availability

All data are publicly freely available in GenBank database (https://www.ncbi.nlm.nih.gov).
